# Supplementary material for: Integrating health services for HIV infection, diabetes and hypertension in sub-Saharan Africa: a cohort study
Source: BMJ Open. 2021 Nov 2;11(11):e053412. doi: 10.1136/bmjopen-2021-053412 (PMC8565555; doi:10.1136/bmjopen-2021-053412)
Supplement: Supplementary data [file bmjopen-2021-053412supp001.pdf]

Appendix

Description of HIV, diabetes and hypertension services provided at MOCCA study facilities.

Country	Facility name	Facility type	Notes *
Tanzania	Amana	Regional Hospital	Separate dedicated clinics run for HIV, diabetes and hypertension
	Shree Hindu Mandal	Hospital (Non Governmental Organisation)	Separate dedicated clinics for HIV, diabetes and hypertension; partial integration already occurring.
	Mwananyamala	Regional Hospital	Separate dedicated clinics run for HIV, diabetes and hypertension
	Mkuranga	District Hospital	Dedicated HIV clinic. Outpatient management of diabetes and hypertension
	Bunju	Dispensary	Dedicated HIV-clinic. No clinic for diabetes or hypertension
Uganda	Kiswa	Health Centre, level III	Dedicated HIV clinic. Separate outpatient screening for diabetes and hypertension patients who are then referred for management to a higher facility as necessary.
	Kisugu	Health Centre, level III	Separate HIV clinic. Separate outpatient screening for diabetes and hypertension patients who are then referred for management to a higher facility as necessary.
	TASO Uganda	Non Governmental Organisation	Providing management and care services to patients living with HIV. Includes management of diabetes and hypertension for patients with HIV-infection.
	Ndejje	Health Centre, level IV	Separate dedicated clinics for HIV, diabetes and hypertension
	Wakiso	Health Centre, level IV	Separate dedicated clinics for HIV, diabetes and hypertension.

* Separate dedicated clinics involved separate waiting and consultation areas, a separate pharmacy, and separate appointments and medical records systems. The patients were seen by dedicated physicians.
