# Supplementary material for: Integrating health services for HIV infection, diabetes and hypertension in sub-Saharan Africa: a cohort study
Source: BMJ Open. 2021 Nov 2;11(11):e053412. doi: 10.1136/bmjopen-2021-053412 (PMC8565555; doi:10.1136/bmjopen-2021-053412)
Supplement: Author's manuscript [file bmjopen-2021-053412.draft_revisions.pdf]

BMJ Open

BMJ Open is committed to open peer review. As part of this commitment we make the peer review history of every article we publish publicly available.

When an article is published we post the peer reviewers' comments and the authors' responses online. We also post the versions of the paper that were used during peer review. These are the versions that the peer review comments apply to.

The versions of the paper that follow are the versions that were submitted during the peer review process. They are not the versions of record or the final published versions. They should not be cited or distributed as the published version of this manuscript.

BMJ Open is an open access journal and the full, final, typeset and author-corrected version of record of the manuscript is available on our site with no access controls, subscription charges or pay-per-view fees (http://bmjopen.bmj.com).

If you have any questions on BMJ Open's open peer review process please email info.bmjopen@bmj.com

BMJ Open

Integrating health services for HIV-infection, diabetes and hypertension in sub Saharan Africa: a cohort study.

Journal:	*BMJ Open*
Manuscript ID	bmjopen-2021-053412
Article Type:	Original research
Date Submitted by the Author:	25-May-2021
Complete List of Authors:	Birungi, Josephine; MRC/UVRI and LSHTM Uganda Research Unit,<br>Kivuyo, Sokoine<br>Garrib, Anupam; Liverpool School of Tropical Medicine, ; Liverpool School of Tropical Medicine<br>Mugenyi, Levi; Infectious Disease Research Collaboration; I-Biostat, Hasselt University<br>Mutungi, Gerald<br>Namakoola, Ivan<br>Mghamba, Janneth<br>Ramaiya, Kaushik<br>Wang, Duolao; Liverpool School of Tropical Medicine<br>Maongezi, Sarah; Ministry of Health, Community Development, Gender, Elderly and Children, Dodoma, Tanzania<br>Musinguzi, Joshua<br>Mugisha , Kenneth<br>Etukoit, Bernard; The AIDS Support Organization<br>Kakande, Ayoub; MRC/UVRI and LSHTM Uganda Research Unit<br>Niessen, Louis; Liverpool School of Tropical Medicine<br>Okebe, Joseph<br>Shiri, Tinevimbo<br>Meshack, Shimwela<br>Lutale, Janet; Muhimbili University of Health and Allied Sciences<br>Gill, Geoff<br>Sewankambo, Nelson; Makerere University College of Health Sciences<br>Smith, Peter<br>Nyrienda, Moffat; Malawi Epidemiology and Intervention Research Unit (MEIRU); London School of Hygiene and Tropical Medicine Faculty of Epidemiology and Public Health<br>Mfinanga, Sayoki<br>Jaffar, Shabbar; Liverpool School of Tropical Medicine, Department of International Public Health
Keywords:	Health policy < HEALTH SERVICES ADMINISTRATION & MANAGEMENT, Hypertension < CARDIOLOGY, DIABETES & ENDOCRINOLOGY, HIV & AIDS < INFECTIOUS DISEASES

For peer review only - http://bmjopen.bmj.com/site/about/guidelines.xhtml

I, the Submitting Author has the right to grant and does grant on behalf of all authors of the Work (as defined in the below author licence), an exclusive licence and/or a non-exclusive licence for contributions from authors who are: i) UK Crown employees; ii) where BMJ has agreed a CC-BY licence shall apply, and/or iii) in accordance with the terms applicable for US Federal Government officers or employees acting as part of their official duties; on a worldwide, perpetual, irrevocable, royalty-free basis to BMJ Publishing Group Ltd ("BMJ") its licensees and where the relevant Journal is co-owned by BMJ to the co-owners of the Journal, to publish the Work in this journal and any other BMJ products and to exploit all rights, as set out in our licence.

The Submitting Author accepts and understands that any supply made under these terms is made by BMJ to the Submitting Author unless you are acting as an employee on behalf of your employer or a postgraduate student of an affiliated institution which is paying any applicable article publishing charge ("APC") for Open Access articles. Where the Submitting Author wishes to make the Work available on an Open Access basis (and intends to pay the relevant APC), the terms of reuse of such Open Access shall be governed by a Creative Commons licence – details of these licences and which Creative Commons licence will apply to this Work are set out in our licence referred to above.

Other than as permitted in any relevant BMJ Author's Self Archiving Policies, I confirm this Work has not been accepted for publication elsewhere, is not being considered for publication elsewhere and does not duplicate material already published. I confirm all authors consent to publication of this Work and authorise the granting of this licence.

For peer review only

Integrating health services for HIV-infection, diabetes and hypertension in sub-Saharan Africa: a cohort study.

CONFIDENTIAL – Please do not circulate

Josephine Birungi [1,2] *, Sokoine L Kivuyo[3] *, Anupam Garrib [4] *, Levicatus Mugenyi [1], Gerald Mutungi [5], Ivan Namakoola [2], Janneth Mghamba [6], Kaushik Ramaiya [7], Duolao Wang [4], Sarah Maongezi, Joshua Musinguzi [5], Kenneth Mugisha [1], Bernard Micheal Etukoit [1], Ayoub Kakande[2], Louis Niessen [4], Joseph Okebe [4], Tinevimbo Shiri [4], Meshack Shimwela [8], Janet Lutale [9], Geoff Gill [4], Nelson K Sewankambo [10], Peter G Smith [11], Moffat J Nyirenda [2, 12], Sayoki Mfinanga [3,4+], Shabbar Jaffar [4+]

* contributed equally (first authors)
+ contributed equally (senior authors)

1. The AIDS Support Organisation, Mulago Hospital Complex, Kampala, Uganda
2. Medical Research Council/Uganda Virus Research Institute & London School of Hygiene and Tropical Medicine (MRC/UVRI & LSHTM), Uganda Research Unit
3. National Institutes for Medical Research, Dar es Salaam, Tanzania
4. Liverpool School of Tropical Medicine, Pembroke Place, Liverpool, L3 5QA
5. Non-Communicable Diseases and AIDS Control Programmes, Ministry of Health, Uganda
6. Directors Office, Ministry of Health, Community Development, Gender, Elderly and Children, Tanzania.
7. Hindu Mandal Hospital, Dar es Salaam, Tanzania
8. Amana Regional Referral Hospital, Dar es Salaam, Tanzania
9. Muhimbili University of Health and Allied Sciences, Dar es Salaam, Tanzania
10. Makerere University College of Health Sciences, Kampala, Uganda
11. MRC Tropical Epidemiology Group, London School of Hygiene & Tropical Medicine, Keppel Street, London WC1E 7HT
12. Medical Research Council / Uganda Virus Research Institute at the London School of Hygiene & Tropical Medicine Uganda Research Unit, Entebbe, Uganda.

Author for Correspondance: Prof Shabbar Jaffar, shabbar.jaffar@lstmed.ac.uk

For peer review only

Author contributions. SJ, MJN, SM, AG, GM, JMg, JMu, SM, JL, GG, NKS and PGS designed the study with support from KR, KM, BE, LN. JB, SLK, IN implemented the study with support from AG, SM, MJN and KR. JM and AK managed the data with support from JB and SLK. JB, SLK, AG wrote the first drafts and SJ led the later drafts. LM did the statistical analysis with support from DW. All authors contributed to various drafts of the paper and saw and approved the final version.

Acknowledgements: We wish to thank Hazel Snell, Erik van Widenfelt, Luis Cuevas, the MOCCA study teams in Tanzania and Uganda and the health facility teams who supported the study and provided patient care, and Drs Mina Nakawuka Ssali and Martha Nabbada from the Ministry of Health in Uganda for their time and support, and Ayubu Masasi, Eric Mgina, Micheal Mubiru and Sebastian Owilla Kidega on data management.

Funding. This research was funded by the National Institute for Health Research (NIHR) (project reference 16/137/87) using UK aid from the UK Government to support global health research. The views expressed in this publication are those of the author(s) and not necessarily those of the NIHR or the UK Department of Health and Social Care.

Conflict of interest. The authors do not declare any conflict of interest

For peer review only

ABSTRACT

Background. HIV, diabetes and hypertension have a high disease burden in sub-Saharan Africa. Health care for these conditions is organised in silos, is inefficient and wastes resources. In a cohort study, management of HIV-infection, diabetes and hypertension was integrated into a single chronic care clinic. We determined the effects of this on retention in care and on clinical indicators.

Methods: In 10 health facilities offering primary care in Dar es Salaam, Tanzania and Kampala, Uganda, integrated care clinics were set up where patients with either HIV-infection, diabetes or hypertension were managed in a single clinic. Participants had the same waiting areas, the same pharmacy, were seen by the same clinical staff in the same consultation rooms, had similar provision of adherence counselling, and similar tracking services if they failed to attend appointments.

Findings.
Between 5th August 2018 and 21st May 2019, 2,640 patients were screened of whom 2,273 (86%) were enrolled into integrated care (832 with HIV-infection alone, 313 with diabetes alone, 546 with hypertension alone and 582 with multiple conditions). They were followed up to 30th January 2020. Overall, 1615/2273 (71.1%) were female and 1689/2266 (74.5%) had been in care for 6 months or more. The proportions of people retained in care were 686/832 (82.5%, 95% CI 79.9%, 85.1%) among those with HIV-infection, 266/313 (85.0%, 95% CI 81.1%, 89.0%) among those with diabetes, 430/546 (78.8%, 95% CI 75.4%, 82.3%) among those with hypertension and 529/582 (90.9%, 95% CI 88.6, 93.3) among those with multimorbidity. Among those with HIV-infection (whether as a single condition or among people with multi-morbidity), the proportion with plasma viral load <100 copies per ml was 423/478 (88.5%).

Conclusion
Integrated management of chronic diseases could improve the control of diabetes and hypertension in Africa, while preserving the gains achieved in HIV control through stand-alone clinics.

Strengths and limitations

- Study is multi-site and is largest ever conducted on integrating chronic care for HIV, diabetes and management in Africa.
- Study was done in close to normal health service conditions and so findings likely to be generalisable.
- The study was intended to determine feasibility and early clinical outcomes. It does not have a comparison group.

For peer review only

BACKGROUND

The prevalence of diabetes and hypertension has risen sharply in Africa, where these conditions are now responsible for around two million premature adult deaths annually [1-6]. There are effective treatments for diabetes and hypertension, but only 10-20% of persons living with these conditions are thought to be in regular care[1, 7, 8], largely because service provision is limited [9-11]. Identifying approaches to increase access to health services for the management of diabetes and hypertension for populations in Africa represents one of the greatest public health challenges of our time.

Health services in most African countries are generally designed for dealing with acute infections and experience of chronic care management is limited [12], except for HIV-infection which, like other chronic diseases, requires life-long management. In Eastern and Southern Africa 83% of people who know they have HIV-infection are in regular care, with 90% of these virally suppressed[13], compared to a retention rate of about 50% a decade or so ago[14, 15]. HIV associated mortality has declined from over 2 million a year to less than half a million a year [13] and hypertension and diabetes will need to achieve similar levels of retention and disease control to achieve the large declines in mortality needed to meet the Sustainable Development Goals[16].

One reason why HIV services have been successful is because they have been organised in stand-alone clinics in most African countries from shortly after the time that effective anti-retroviral drugs became available. People living with HIV-infection are managed in separate primary care clinics, with separate drug and diagnostic procurement channels and a separate funding stream. Now that the burden of other chronic conditions is a rapidly increasing public health problem, how should African health systems organise their services? Separate stand-alone clinics for each chronic condition are unlikely to be affordable for governments and hugely problematic for patients with multiple conditions.

Managing multiple chronic conditions in the same clinics could reduce the duplication of health care resources and, crucially, could enable the learning acquired by HIV treatment programmes to be applied to the control of other chronic conditions. However, there is little evidence to support this in public health facilities [17, 18]. Integrated management could deter people with diabetes and hypertension in seeking care because of the stigma associated with HIV-infection. There is also a danger, that expanding the focus of health care provision in relatively weak health systems, could put at risk the gains achieved by HIV control programmes. In Tanzania and Uganda, working in close collaboration with policy makers and senior disease control managers, we conducted a study of the integrated care for HIV-infection, diabetes and hypertension and evaluated outcomes in terms of retention in care and clinical indicators.

Methods

Study design and setting.

For peer review only - http://bmjopen.bmj.com/site/about/guidelines.xhtml

We conducted a single arm intervention study, known as the MOCCA study (Management of Chronic Conditions in Africa). We provided integrated care at 10 health facilities that were offering primary health care services, 5 in the Dar es Salaam region in Tanzania and 5 in and around Kampala, Uganda. Appendix 1 describes the health facilities included.

The facilities were serving largely urban and peri-urban populations and chosen purposely. Eight were government run and 2 were run by non-government organisations. They were chosen on the basis of their infrastructure [19, 20], ranging from regional hospitals through to smaller health centres and dispensaries. The larger facilities operated stand-alone separate clinics for diabetes and hypertension, while in the small facilities, patients with diabetes or hypertension were usually seen in the outpatient clinic. Each facility had a HIV clinic run separately from the rest of the clinical services. Patients attending HIV services had their own reception and waiting area, were seen by dedicated clinical staff, and had a separate pharmacy. Medical records, track and trace procedures, and appointments also all differed from systems employed in other clinics in the facility.

What was integrated care? Integration involved the formation of a single clinic for the management of patients with HIV-infection, diabetes or hypertension or combinations of these conditions. All patients were seen by the same clinical staff. They shared a single reception, waiting areas and pharmacy. For patients with diabetes or hypertension, systems for tracking and follow up of patients, recording of clinical notes, counselling, appointments and registrations were mostly set up from new and aligned with those available for people living with HIV, such that all patients were managed in a similar way regardless of their condition.

Stakeholder engagement: The concept of integrated care was developed in partnership with senior policy makers. It was a novel approach and so needed substantial stakeholder engagement. In each country, we held planning meetings with the Ministry of Health and non-governmental partners at national and local level, with senior clinicians and hospital managers and with patient and community representatives. National steering committees were formed, comprising representatives from these stakeholders together with the researchers. We also formed an international steering committee, that included 4 senior researchers and 4 independent researchers, which had oversight of the programme. Each committee usually met twice a year.

How was integrated care implemented?

We had an observation period for 1-6 months prior to implementing integrated care in 6 of the facilities. In the remaining 4 facilities, implementation was done straightaway without an observation period as by then we considered we had sufficient understanding of the health care delivery to implement integrated care without a prior period of observation. In Uganda the first person received integrated care on the 5th October 2018 and recruitment finished on the 5th of April 2019. The final follow-up was on the 20th December 2019. In Tanzania, the first person received integrated care on 20th February 2019. Recruitment finished on 23rd June 2019. The final follow-up was on 30 January 2020.

During the observation period, we conducted assessments of each facility using a modified WHO Service Availability and Readiness Assessment (SARA) tool and reviewed routine data

For peer review only - http://bmjopen.bmj.com/site/about/guidelines.xhtml

from the Health Management Information Systems (District Health Information System 2). This provided us with knowledge on the clinical infrastructure, availability of medicines, frequency and nature of patient attendance for assessments.

The integrated care clinic was set up to run separately as a stand-alone clinic at each facility so that patients who did not want integrated care would be able to continue with usual clinics. Organisation of the integrated care clinic, including the frequency of clinic days, was done by health facility managers. Where possible the integrated clinic was run on a different day from the HIV and hypertension or diabetes standalone clinics. In those facilities where this was not possible, it was run in a separate room away from the main standalone clinics. The integrated clinic was held initially on one day a week at all facilities and later increased to 2 days a week as the numbers of recruited participants increased. Clinical management was done by health care staff employed by the facilities and in accordance with local clinical practice and national guidelines.

The health care staff received refresher training in all 3 conditions to ensure a common level of understanding of clinical management. The training comprised a combination of two days of classroom sessions and on-the-job training in which doctors specialised in HIV and in non-communicable disease management conducted joint clinics for up to one month until they felt comfortable to manage all three conditions. Classroom training included role play of different scenarios. In Uganda, this was done mostly by a certified national trainer while in Tanzania, it was led by a senior physician from a non-governmental organisation run hospital.

Counselling was generally not available for diabetes and hypertension management in either country prior to the study but was available routinely for patients with HIV-infection. We therefore provided training to nurses and counsellors on diabetes and hypertension, focussed on the need for high treatment adherence to prevent complications. It comprised one day of classroom training followed by on-the-job training for up to 4 weeks, as done for clinicians, and mentorship.

Prior to the study, HIV clinics had a system of tracking patients who did not attend a scheduled appointment. This generally involved contacting patients by phone if they missed an appointment, to determine the reason for the missed appointment and to reschedule. Such practices were not in place for people with either diabetes or hypertension. Therefore, we extended the system of tracking patients to cover all 3 conditions.

All health facility staff received training on the protocol and research methods. This was approximately one-day of classroom training, followed by on-the-job training.

Data collection: At each clinic, the research team employed one data clerk and one research nurse to inform patients about the research, collect research data and take informed consent. In addition, small amounts of salary (salary top-ups) were provided to selected facility staff working with study participants and to the head nurse responsible for the facility operations including overseeing the logistical set up of integrated care activities. Research staff worked alongside the health facility staff.

For peer review only - http://bmjopen.bmj.com/site/about/guidelines.xhtml

Selection of participants. In each disease-specific clinic a health talk was given to introduce the study. The number of patients with diabetes or with multiple conditions was very few. Therefore, all persons presenting with diabetes or with two or all 3 of our target conditions were invited consecutively to join the study. Both existing and newly diagnosed patients were enrolled. In the larger facilities (n=3, all in Tanzania), the number of people in care with HIV-infection or with hypertension was large and so we sampled systematically (usually every 20th patient) until the clinic ended for the day. In the remaining clinics, where patient load was lower and it was difficult to ascertain the order that patients had come to the facility, we enrolled patients who approached study staff to find out more, or clinicians approached patients and invited them to be screened for the study. On most clinic days, patients who had recently arrived at the facility came forward for enrolment, and those who had nearly completed their consultations for the day and were close to leaving the facility for home, were reluctant to come forward.

Provision of medicines for hypertension and diabetes

Before the study started, we recognised that supply of medicines for diabetes and hypertension was erratic in both countries. In Uganda, medicines for these conditions were normally provided free but shortages were common. In Tanzania, most patients were required to purchase medicines, and some had health insurance. We have discussed elsewhere the ethical challenges of conducting research of this kind in situations of limited medicines supply[21].

To ensure a reliable supply of drugs, we purchased back-up supplies of basic drugs for diabetes and hypertension in both Tanzania and Uganda and supplemented patient supplies when the health service stock ran out. The medicines supplied were predominantly first line drugs and differed between countries depending on what was needed.

Due to the limited supply of medicines for diabetes and hypertension in Uganda, patients at one of the study facilities had organised themselves into a group that met and pooled funds on a monthly basis. These funds were used to purchase the medicines when the health facility ran out of stock. At this facility, we offered minimal buffer supplies compared to other facilities.

Measurements.

Retention was assessed through routine attendance at clinics. Patients who failed to attend an appointment were phoned to establish reasons for not attending and to reschedule. Three attempts were made to contact the patient by phone in the month following non-attendance at an appointment. A patient was regarded as lost to follow up if they had not attended an appointment in 6 months.

Blood pressure was measured using the Omron M6 comfort (OMRON Healthcare, Japan) in Uganda and in 3 of the facilities in Tanzania; Suresign SLD3-107 (CIGA Healthcare Ltd, UK) was used in the remaining facilities. We measured blood pressure at 3 time points in the sitting position, 5 minutes apart, and took the average of the three readings as the pressure. Hypertension was defined as a systolic pressure ≥140mmHg and/or a diastolic pressure ≥90mmHg and/or use of antihypertensive medication.

For peer review only

Plasma viral load testing was done by the Central Public Health laboratories in Tanzania and Uganda using the Cobas 8800 system (Roche). In Uganda, if the viral load result from the central laboratory was not available or was more than 6 months old, a test was done at the MRC/UVRI/LSHTM Uganda laboratory using Cobas Ampliprep. In both countries HbA1c testing was not routinely available in the public health system, and this was done by the study. In Uganda, HbA1c was tested by Cobas 6000 (Roche, Switzerland) at the MRC/UVRI/LSHTM Uganda laboratory, and in Tanzania at the Shree Hindu Mandal Hospital laboratory using a Siemens DCA Vantage (Siemens Healthcare Diagnostics, Germany). Fasting blood glucose was done using a point of care test in Tanzania (GlucoDr. Auto AGM-4000 and (all Medicus Co, Republic of Korea) and in Uganda Contour Plus (Bayer, Germany).

In Tanzania weight and height were measured using SECA RGZ-160 (SECA, GmbH & Co, Germany). In Uganda we used an ADE spring scale (kg, 1-150) for weight measurement and SECA 213 portable stadiometer (SECA, German) for height measurement. Weight and height were measured to the nearest ±0.1 kg and ±0.1 cm, respectively, and body mass index (BMI) was calculated as weight/height^2 (kg/m^2).

Patient and Public involvement
Patient representatives attended our planning meetings and contributed to the design of the study and other aspects of the research, such as its implementation. We set up steering committees in both Tanzania and Uganda, which comprised researchers, policy makers and had patient representatives to oversee the implementation. We also held investigator meetings involving all of the partners. These included patient representatives.

Statistical analysis
The measure of retention used was defined as number in care at study end, calculated as the number enrolled into integrated care minus any deaths, losses-to-follow-up, transfers away from the health facility and withdrawals of consent.

Categorical variables were summarised using frequencies and proportions and compared using Chi-square tests. Continuous variables were summarised using means or medians and compared by a t-test or a Wilcoxon test, as appropriate. Generalized Estimating Equations (GEE) were used to account for clustering at the health facility level. Kaplan Meier curves and Cox regression with frailties (to adjust for clustering) were used to analyse retention times. The time was calculated as number of months from first attendance at the integrated clinic to the earliest of the end of the study, loss to follow up, death, withdrawal, or transfer-out. The analysis was done using STATA version 15.

Ethics clearance
The study received ethical clearance from TASO Research Ethics Committee and Uganda National Council for Science and Technology (UNCST) in Uganda (Reference TASO REC/015/18-UG-REC-009), National Health Research Ethics Review Sub-Committee in Tanzania (Reference NIMR/HQ/R.8a/Vol. IX/2793), and the Liverpool School of Tropical Medicine in the UK (Reference 18-044).

For peer review only - http://bmjopen.bmj.com/site/about/guidelines.xhtml

RESULTS

Figure 1 shows the consort chart. Of 2640 patients assessed for eligibility, 102 (3.9%) did not meet eligibility criteria (34 were very sick, 50 were planning to move out of the area, 13 were pregnant and receiving care from antenatal care services, 5 other reasons), 19 (0.7%) declined and gave no reason, 51(1.9%) wanted more time to decide (mostly to discuss with their family), 34 (1.3%) were approached just as they had finished consultation and were leaving the clinic and 18 (0.7%) gave other reasons (mostly that they needed to be in specialist care).

Thus, of all those initially assessed for eligibility 91.5% (2416/2640) were invited to join integrated care. This invitation was given at their current clinics and they were asked to return at their next visit to the integrated care clinic. Six percent (143/2416) did not return and thus 2,273/2640 (86.1%) were enrolled into integrated care, 927 in Tanzania and 1346 in Uganda.

Table 1 shows the characteristics of the participants at the start of integrated care, for each clinical condition. Overall, 74.5% (1689/2266) reported that they had been in care for 6-months or more prior to the start of the study. For people with multiple conditions, previous time in care was substantially greater than for those with single conditions. Overall, 71.1% (1615/2273) of those included in the study were female.

Median age varied substantially between the different conditions, with that for those living with HIV-infection being more than 10 years younger than participants in the other groups (p=0.0001, Kruskal Wallis test). Obesity was more common among participants with diabetes, hypertension or multimorbidity than with HIV-infection (504/1441 (35.0%) versus 116/832 (13.9%), risk ratio adjusted for clustering: 2.50, 95% CI: 1.88, 3.31, p<0.001). It was also substantially higher in women than in men (554/1615 (34.3%) vs 66/658 (10.0%), adjusted risk ratio: 3.40, 95% CI: 2.63– 4.41, p<0.001).

Table 2 shows the biomedical indicators as measured at baseline on the day of integration or within the previous 6 months. The majority did not reach target blood pressure and glycaemia control levels, whether they had a single condition or multimorbidity. When participants who reported that they had been in care for less than 6 months (i.e. patients who had newly started therapy) were excluded, 509/882 (57.7%) of those with hypertension had a blood pressure ≥140/90 mmHg, 355/508 (69.9%) of those with diabetes had a fasting blood glucose ≥7.0 mmol/L and 72/438 (16.4%) of those with HIV-infection had a plasma viral load ≥100 copies per ml.

The proportions alive and retained in care were high among all participants (Table 3). The overall proportion alive and in care among participants with a single condition was 1382/1691 (81.7%, 95% CI: 79.9% - 83.6%), which did not differ significantly from the proportion with multimorbidity (adjusted risk ratio 0.91, 95% CI: 0.82, 1.00, p=0.061).

Figure 2 shows the survival probability curves of being alive and in care over time. After adjusting for clustering, the probability (95% CI) of being retained and alive in care at 12

For peer review only - http://bmjopen.bmj.com/site/about/guidelines.xhtml

months was 0.74 (0.66 – 0.80) among participants with HIV alone, 0.74 (0.66– 0.81) among those with diabetes alone, 0.70 (0.64 – 0.76) among those with hypertension alone and 0.84 (0.79 – 0.88) among those with multimorbidity (p=0.008).

Table 4 shows the biomedical indicators of participants at the final follow-up. The proportions of patients living with HIV-infection with viral suppression were high, but the proportions of those with hypertension achieving blood pressure less than 140/90 mmHg and those with diabetes achieving glycaemia <7 mmol/L were substantially lower. However, blood pressure, fasting glucose and plasma viral load were all better controlled at study end than at baseline (Table 5), even when participants who had been in care for less than 6 months, and would have been newly started on therapy were excluded. After adjusting for clustering, among patients with HIV, the proportion (95% CI) with plasma viral load of <100 copies per ml was 88.5% (85.7%-91.4%) and those with plasma viral load <1000 copies per ml was 96.0% (94.3%-97.8%).

DISCUSSION

This study shows that integrating care for HIV-infection, diabetes and hypertension achieved high levels of retention in care for people living with diabetes or hypertension in Africa. Crucially, this improvement did not appear to be at the expense of HIV control, as retention in all 3 groups of people with single conditions was similar, and the proportion with an HIV viral suppression of less than a 100 copies per ml was close to 90%.

Thus, our study suggests that an integrated approach could achieve excellent retention for diabetes and hypertension in an integrated clinic, as has been achieved for people living with HIV-infection where retention exceeds 80% today, up from about 50% a decade or so ago[13-15].

The retention rate for people with diabetes or hypertension observed in our study was higher than that published from some other studies, which involved studies of single diseases, including those where diagnostic and treatment services were enhanced and, for example, treatments were provided for free[22-24]. In our study, we put in place basic measures to support participants with diabetes and hypertension including minimal access to basic medicines, counselling and telephone reminders for those who missed appointments. However, these measures were no more than those available to people in care with HIV-infection and no more than those available to people in research studies of hypertension and diabetes. No financial or other incentives were provided in our study and participants were managed by routine health care staff. Analysis of the costs of this package will be done separately but generic medicines for diabetes and hypertension and associated monitoring costs are substantially less than those for HIV-infection and so we anticipate that the model will be highly cost-effective.

In a large study in Malawi conducted by members of our team almost a decade ago, people identified with diabetes and hypertension in the community were referred into care with a dedicated medical officer and access to a free and reliable supply of medicines and diagnostics. However, at 12 months retention in care was 27% , suggesting that control of

For peer review only - http://bmjopen.bmj.com/site/about/guidelines.xhtml

diabetes and hypertension when these conditions are identified through routine screening (i.e. are asymptomatic) might be a challenge[23, 25]. A study from Kwazulu-Natal in South Africa reported similar findings [26]. However, other studies that provided a good level of care and that have involved patients who know they have diabetes and hypertension, have also struggled to achieve good levels of retention[22].

Why did our study achieve high retention? For people with multiple conditions, a huge advantage of integrated care is that they only need to attend one clinic appointment, but in our study retention was also high for people with single conditions. It is possible that when services were integrated and the lessons learned in managing HIV-infection with respect to counselling, treatment adherence and the provision of a more streamlined service, were applied by health care staff to diabetes and hypertension management, patients responded to the changes in service delivery. The mixing of patients with HIV-infection with those living with diabetes and hypertension may also have led to the sharing of experiences of living with a long-term condition, which could have brought further benefit.

We knew that integration of services would involve expansion of care responsibilities of health facility staff, and feared that this would adversely impact HIV care outcomes [17, 27, 28]. However, this did not happen. HIV viral suppression rates were high at enrolment and were slightly higher at the end of the study. There is an increasing body of literature on integrating screening, diagnosis and treatment for non-communicable diseases into established HIV services.[17, 29, 30] As far as we are aware, ours is the first study to test integration of services fully so that any person with one or more of the 3 target conditions could be managed in one clinic[29, 31].

We had feared that stigma may put off patients with diabetes and hypertension from attending a joint service with patients living with HIV. However, none of the patients who declined to join cited stigma. It is possible that the 41 patients who declined without giving a reason or who deferred their decision, did so because of the stigma. Nonetheless, these numbers of refusals are relatively small, and very few study participants withdrew over time. HIV has a special status in almost all African countries, which may have contributed to the stigma associated with this disease. Our study suggests that HIV-infection can be managed in the same space as other conditions – i.e. that HIV can be considered like any other disease or condition – and this will not make a difference to the outcome for the patient living with HIV-infection.

Although we achieved high rates of retention, and blood pressure and glycaemia control improved over time, a high percentage of those with diabetes or hypertension continued to have uncontrolled blood pressure and uncontrolled glycaemia at the end of the study, in contrast to the situation with HIV for which the vast majority maintained excellent control of viraemia. In the case of diabetes, this is complicated by a lack of access to adequate monitoring. Access to HbA1c testing was a challenge we faced in this study, and is a challenge across Africa[32]. The next step in this story is to identify and test interventions that improve blood pressure and diabetes control (among people in care) and take this control to levels achieved by HIV programmes in order to drive down mortality from non-communicable diseases.

For peer review only - http://bmjopen.bmj.com/site/about/guidelines.xhtml

Large numbers of people in high income countries are now living with multiple conditions – so called multimorbidity - and this number is expected to continue to rise sharply in Africa, if it has not done so. In our study, individuals with more than one condition, who would normally attend multiple different clinics, appeared to have a higher retention in care than people with a single condition but their clinical indicators at study end were no different from those with a single condition. While it is not surprising that their retention should be high, the lack of superiority in their clinical indicators from those with a single condition, suggest that greater efforts will be needed to improve clinical outcomes.

Although the evidence generated by our study is much greater than that available to most policy makers, and separate clinics for each chronic condition is not sustainable for Ministries of Health, changes to health policy that would affect tens of millions of people living with chronic conditions, requires robust evidence. Our study was only intended to be a pilot study and did not have a comparison group and its size was limited. Evidence is needed from large randomised trials with control groups[17], and with replication in multiple settings and over a longer duration of follow-up. We also need more evidence on why integrated care works, particularly for those with single conditions.

Studies that involve re-organisation of care provision represent huge change and challenges for health services, communities and patients. They are not possible without partnerships based on an ethos of equality and openness and without the building of trust between researchers and these stakeholders. It is essential that research of this nature allows time and resource for the working together of different groups of researchers, health care providers and public health authorities, patients and communities.

For peer review only

Table 1. Baseline characteristics of patients studied.

		HIV only	Diabetes only	Hypertension only	Multiple conditions
**Number recruited into integrated care**					
	**Total**	**832**	**313**	**546**	**582**
	In Tanzania	352	164	160	251
	In Uganda	480	149	386	331
**Type of health facility**					
	Large public facilities	235	81	153	184
	Small public facilities	409	155	282	217
	NGO facilities	188	77	111	181
**Time in care prior to enrolment, number (%) ***					
	< 6 months	310 (37.4)	63 (20.3)	165 (30.3)	39 (6.7)
	6-11 months	41 (5.0)	28 (9.0)	42 (7.7)	22 (3.8)
	12-59 months	167 (20.1)	98 (31.6)	172 (31.6)	135 (23.2)
	>=60 months	311 (37.5)	121 (39.0)	166 (30.5)	386 (66.3)
**Median (range) months in care**		26.1 (0.0 – 343.4)	38.3 (0.0 – 431.6)	26.4 (0.0 – 566.2)	106 (0.0 – 534.8)
**Female, number (%)**		571 (68.6)	208 (66.5)	399 (73.1)	437 (75.1)
**Median age, (range)**		38 (18 – 78)	49 (19 – 77)	56 (27 – 86)	55 (21 – 85)
**BMI, number (%)**					
	<25	531 (63.8)	118 (37.7)	175 (32.1)	163 (28.0)
	25-29.9	185 (22.2)	98 (31.3)	181 (33.2)	202 (34.7)
	≥30	116 (13.9)	97 (31.0)	190 (34.8)	217 (37.3)
**Median BMI (range) in men**		21.1 (14.8 – 35.6)	24.3 (15.1 – 37.0)	25.1 (16.7 – 41.6)	26.0 (17.0 – 53.1)
**Median BMI (range) in women**		24.4 (13.2 – 48.9)	28.5 (15.4 – 48.2)	28.8 (16.0 – 65.2)	28.8 (15.8 – 57.9)

NOTES

* missing for 7 participants (3 with HIV-infection, 3 diabetes and 1 hypertension).

For peer review only

Table 2. Biomedical indicators at baseline.

	Patients who have a single condition occurring alone	The condition occurs with other conditions, causing multimorbidity (only among those with the condition)	Both combined
**AMONGST PATIENTS WITH HYPERTENSION**			
**Blood pressure (mmHg), number (%)**	**N=546**	**N=539**	**N=1085**
**≥140/90**	342 (62.6)	327 (60.7)	669 (61.7)
**<140/90**	204 (37.4)	212 (39.3)	416 (38.3)
**≥180/120**	42 (7.7)	64 (11.9)	106 (9.8)
**<180/120**	504 (92.3)	475 (88.1)	979 (90.2)
**Systolic BP mmHg, mean (range)**	144.4 (83.0 – 257.7)	145.1 (68.7 – 271.0)	144.7 (68.7 – 271.0)
**Diastolic BP mmHg, mean (range)**	90.2 (43.3 – 163.0)	90.3 (54.7 – 167.3)	90.2 (43.3 – 167.3)
**AMONGST PATIENTS WITH DIABETES**			
**Fasting glucose (mmol/L), number (%)**	**N=228**	**N=281**	**N=509**
<6.1	34 (14.9)	62 (22.1)	96 (18.9)
6.1–6.9	17 (7.5)	32 (11.4)	49 (9.6)
≥7.0	177 (77.6)	187 (66.6)	364 (71.5)
**Fasting glucose, mean (range) mmol/L**	11.5 (2.9 – 29.3)	9.8 (2.3 – 28.2)	10.5 (2.3 – 29.3)
**HbA1c, number (%) ***	**N=124**	**N=206**	**N=330**
(<6.0%	4 (3.2)	25 (12.1)	29 (8.8)
6.0–6.4%	7 (5.7)	22 (10.7)	29 (8.8)
≥6.5%	113 (91.1)	159 (77.2)	272 (82.4)
**HbA1c, mean (range) percent**	10.1 (4.8 – 16.9)	8.8 (4.8 – 16.5)	9.3 (4.8 – 16.9)
**AMONGST PATIENTS LIVING WITH HIV**			
**HIV plasma viral load, number (%) ***	**N= 493**	**N= 153**	**N= 646**
<100 copies/ml	306 (62.1)	131 (85.6)	437 (67.7)
100-999 copies/ml	68 (13.8)	14 (9.2)	82 (12.7)
≥1000 copies/ml	119 (24.1)	8 (5.2)	127 (19.7)

* measured at any time within the previous 6 months. Overall for the proportion with plasma viral load <100 copies/ml, the 95% CI was 64.1%, 71.4% and 80.3% (95% CI 77.3%-83.5%) had a plasma viral load < 1000 copies per ml.

Note: Blood pressure was measured at baseline by research staff among 1,187/1188 (99.9%) participants who were not known previously to be hypertensive. Of these, the proportion who were detected with a blood pressure of 140/90 mmHg or higher was 80/831 (9.6%) among those with HIV-infection alone, 47/313 (15.0%) among those with diabetes alone and 6/43 (14.0%) among those with HIV-infection and diabetes. Of note, among all HIV-infected participants, blood pressure was 180/120 mmHg or higher among 45/1126 (4.0%).

For peer review only

Table 3. Retention in integrated care and clinical outcomes according to condition.

**Overall**	**HIV only**	**Diabetes only**	**Hypertension only**	**Multiple conditions**
**Number enrolled into integration**	**832**	**313**	**546**	**582**
Median follow-up (inter-quartile range)	8.2 (6.9 – 10.4)	8.0 (6.9 – 9.4)	8.2 (6.9 – 10.1)	8.2 (7.2 – 9.8)
Retained in care to the end of the study n [% (95%CI)]*	n. 686 82.5 (79.9 –85.1)	n. 266 85.0 (81.1 – 89.0)	n. 430 78.8 (75.4 – 82.3)	n. 529 90.9 (88.6–93.3)
Rate per 100 person years (95% CI)*	75.0 (70.6, 78.7)	77.7 (70.4, 83.3)	69.9 (63.5, 74.6)	87.1 (83.2, 90.2)

* adjusted for clustering at health facility level

Notes: 85 persons were newly diagnosed with multiple conditions over the course of follow-up. Of participants with HIV alone, 5 were diagnosed with diabetes and 33 with hypertension. Of those with diabetes, 3 tested positive for HIV and 23 were diagnosed with hypertension. Of those with hypertension, 1 tested positive for HIV and 9 were diagnosed with diabetes. Eleven persons with dual conditions were diagnosed with a 3rd condition.

For peer review only

Table 4. Biomedical indicators at study end.

		Patients who have a single condition occurring alone	The condition occurs with other conditions, causing multimorbidity	Both combined
**Blood pressure (mmHg), number (%)**		**N=415**	**N=469**	**N=884**
	≥140/90	183 (44.1)	225 (48.0)	408 (46.2)
	<140/90	232 (55.9)	244 (51.2)	476 (53.9)
	≥180/120	7 (1.7)	25 (5.3)	32 (3.6)
	<180/120	408 (98.3)	444 (94.7)	852 (96.4)
**Systolic BP mmHg, mean (range)**		133.9 (26.0 – 228)	136.7 (90.0 – 227)	135.4 (26 – 228)
**Diastolic BP mmHg, mean (range)**		83.9 (53 – 130)	85.6 (44 – 185)	84.8 (44 – 185)
**Fasting glucose (mmol/L), number (%)**		**N=189**	**N=242**	**N=431**
	<6.1	36 (19.1)	69 (28.5)	105 (24.4)
	6.1–6.9	28 (14.8)	33 (13.6)	61 (14.2)
	≥7.0	125 (66.1)	140 (57.9)	265 (61.5)
**Fasting glucose, mean (range) mmol/L**		9.7 (3.6 – 28.5)	8.3 (3.6 – 19.8)	8.9 (3.6 – 28.5)
**HbA1c, number (%).**		**N=84**	**N=140**	**N=224**
	(<6%	17 (20.2)	31 (22.1)	48 (21.4)
	6.0-6.4%	4 (4.8)	15 (10.7)	19 (8.5)
	≥6.5%	63 (75.0)	94 (67.1)	157 (70.1)
**HbA1c, mean (range) percent**		7.6 (0.6 – 13.5)	7.3 (0.6 – 15.9)	7.4 (0.6 – 15.9)
**HIV plasma viral load, number (%) ***		**N=322**	**N=156**	**N=478**
	<100 copies/ml	278 (86.3)	145 (93.0)	423 (88.5)
	100-999 copies/ml	27 (8.4)	9 (5.8)	36 (7.5)
	>=1000 copies/ml	17 (5.3)	2 (1.3)	19 (4.0)

* The 95% CI for plasma viral load<100 copies/ml for both groups combined was 85.7, 91.4%). The proportion with plasma viral load<1000 copies per ml was 96.0% (95% CI 94.3%, 97.8%).

Note: 80 persons were newly diagnosed with 2nd or 3rd conditions over the course of the follow-up (see Table 3). Their final follow-up indicators are not included in the analyses above as these patients may have been newly started on treatment and therefore will not have had time to stabilise.

For peer review only

Table 5. Risk differences (95% CI) between baseline and study end among participants living with hypertension, diabetes or HIV-infection.

Clinical condition (either alone or alongside other conditions)	All participants	Excluding participants in care for less 6 months at enrolment
Raised blood pressure (≥140/90 mmHg)	14.8%, (10.5%, 19.2%) (p<0.001)	10.5%, (5.7%, 15.3%) p<0.001).
Raised fasting glucose (≥7 mmol/L)	8.4%, (2.4%, 14.3%) p=0.006)	7.4% (1.0%, 13.7%) p=0.024)
Lack of viral suppression (plasma viral load ≥ 100 copies per ml)	20.8%, (16.0%, 25.5%) p<0.001).	7.3% (2.7%, 11.9%) p=0.002)

Notes: the risk difference is calculated as risk estimate at baseline minus risk estimate at study end.

For peer review only

References

1. Atun, R., J.I. Davies, E.A.M. Gale, T. Barnighausen, D. Beran, A.P. Kengne, N.S. Levitt, F.W. Mangugu, M.J. Nyirenda, G.D. Ogle, K. Ramaiya, N.K. Sewankambo, E. Sobngwi, S. Tesfaye, J.S. Yudkin, S. Basu, C. Bommer, E. Heesemann, J. Manne-Goehler, I. Postolovska, V. Sagalova, S. Vollmer, Z.G. Abbas, B. Ammon, M.T. Angamo, A. Annamreddi, A. Awasthi, S. Besancon, S. Bhadriraju, A. Binagwaho, P.I. Burgess, M.J. Burton, J. Chai, F.P. Chilunga, P. Chipendo, A. Conn, D.R. Joel, A.W. Eagan, C. Gishoma, J. Ho, S. Jong, S.S. Kakarmath, Y. Khan, R. Kharel, M.A. Kyle, S.C. Lee, A. Lichtman, C.P. Malm, M.N. Mbaye, M.A. Muhimpundu, B.M. Mwagomba, K.J. Mwangi, M. Nair, S.P. Niyonsenga, B. Njuguna, O.L.O. Okafor, O. Okunade, P.H. Park, S.D. Pastakia, C. Pekny, A. Reja, C.N. Rotimi, S. Rwunganira, D. Sando, G. Sarriera, A. Sharma, A. Sidibe, E.S. Siraj, A.S. Syed, K. Van Acker, and M. Werfalli, *Diabetes in sub-Saharan Africa: from clinical care to health policy.* Lancet Diabetes Endocrinol, 2017. **5**(8): p. 622-667.
2. Bosu, W.K., S.T. Reilly, J.M.K. Aheto, and E. Zucchelli, *Hypertension in older adults in Africa: A systematic review and meta-analysis.* PLoS One, 2019. **14**(4): p. e0214934.
3. Guwatudde, D., J. Nankya-Mutyoba, R. Kalyesubula, C. Laurence, C. Adebamowo, I. Ajayi, F. Bajunirwe, M. Njelekela, F.S. Chiwanga, T. Reid, J. Volmink, H.O. Adami, M.D. Holmes, and S. Dalal, *The burden of hypertension in sub-Saharan Africa: a four-country cross sectional study.* BMC Public Health, 2015. **15**: p. 1211.
4. Mills, K.T., J.D. Bundy, T.N. Kelly, J.E. Reed, P.M. Kearney, K. Reynolds, J. Chen, and J. He, *Global Disparities of Hypertension Prevalence and Control: A Systematic Analysis of Population-Based Studies From 90 Countries.* Circulation, 2016. **134**(6): p. 441-50.
5. Organisation, W.H., *Descrption status report on non-communicable diseases 2010.* 2011, 2010(Geneva, Switzerland).
6. Sarki, A.M., C.U. Nduka, S. Stranges, N.B. Kandala, and O.A. Uthman, *Prevalence of Hypertension in Low- and Middle-Income Countries: A Systematic Review and Meta-Analysis.* Medicine (Baltimore), 2015. **94**(50): p. e1959.
7. Addo, J., L. Smeeth, and D.A. Leon, *Hypertension in sub-saharan Africa: a systematic review.* Hypertension, 2007. **50**(6): p. 1012-8.
8. Geldsetzer, P., J. Manne-Goehler, M.E. Marcus, C. Ebert, Z. Zhumadilov, C.S. Wesseh, L. Tsabedze, A. Supiyev, L. Sturua, S.K. Bahendeka, A.M. Sibai, S. Quesnel-Crooks, B. Norov, K.J. Mwangi, O. Mwalim, R. Wong-McClure, M.T. Mayige, J.S. Martins, N. Lunet, D. Labadarios, K.B. Karki, G.B. Kagaruki, J.M.A. Jorgensen, N.C. Hwalla, D. Houinato, C. Houehanou, M. Msaidie, D. Guwatudde, M.S. Gurung, G. Gathecha, M. Dorobantu, A. Damasceno, P. Bovet, B.W. Bicaba, K.K. Aryal, G. Andall-Brereton, K. Agoudavi, A. Stokes, J.I. Davies, T. Barnighausen, R. Atun, S. Vollmer, and L.M. Jaacks, *The state of hypertension care in 44 low-income and middle-income countries: a cross-sectional study of nationally representative individual-level data from 1.1 million adults.* Lancet, 2019. **394**(10199): p. 652-662.
9. Katende, D., G. Mutungi, K. Baisley, S. Biraro, E. Ikoona, R. Peck, L. Smeeth, R. Hayes, P. Munderi, and H. Grosskurth, *Readiness of Ugandan health services for the management of outpatients with chronic diseases.* Trop Med Int Health, 2015. **20**(10): p. 1385-95.
10. Peck, R., J. Mghamba, F. Vanobberghen, B. Kavishe, V. Rugarabamu, L. Smeeth, R. Hayes, H. Grosskurth, and S. Kapiga, *Preparedness of Tanzanian health facilities for*

outpatient primary care of hypertension and diabetes: a cross-sectional survey. Lancet Glob Health, 2014. **2**(5): p. e285-92.

11. Manne-Goehler, J., R. Atun, A. Stokes, A. Goehler, D. Houinato, C. Houehanou, M.M. Hambou, B.L. Mbenza, E. Sobngwi, N. Balde, J.K. Mwangi, G. Gathecha, P.W. Ngugi, C.S. Wesseh, A. Damasceno, N. Lunet, P. Bovet, D. Labadarios, K. Zuma, M. Mayige, G. Kagaruki, K. Ramaiya, K. Agoudavi, D. Guwatudde, S.K. Bahendeka, G. Mutungi, P. Geldsetzer, N.S. Levitt, J.A. Salomon, J.S. Yudkin, S. Vollmer, and T. Barnighausen, *Diabetes diagnosis and care in sub-Saharan Africa: pooled analysis of individual data from 12 countries.* Lancet Diabetes Endocrinol, 2016. **4**(11): p. 903-912.
12. Garrib, A., J. Birungi, S. Lesikari, I. Namakoola, T. Njim, L. Cuevas, L. Niessen, K. Mugisha, G. Mutungi, J. Mghamba, K. Ramaiya, S. Jaffar, S. Mfinanga, and M. Nyirenda, *Integrated care for human immunodeficiency virus, diabetes and hypertension in Africa.* Trans R Soc Trop Med Hyg, 2019. **113**(12): p. 809-812.
13. UNAIDS, *UNAIDS Data 2020*. 2020, UNAIDS: Geneva, Switzerland.
14. Rosen, S., M.P. Fox, and C.J. Gill, *Patient retention in antiretroviral therapy programs in sub-Saharan Africa: a systematic review.* PLoS Med, 2007. **4**(10): p. e298.
15. Rosen, S. and M.P. Fox, *Retention in HIV care between testing and treatment in sub-Saharan Africa: a systematic review.* PLoS Med, 2011. **8**(7): p. e1001056.
16. collaborators, N.C.D.C., *NCD Countdown 2030: pathways to achieving Sustainable Development Goal target 3.4.* Lancet, 2020. **396**(10255): p. 918-934.
17. Haldane, V., H. Legido-Quigley, F.L.H. Chuah, L. Sigfrid, G. Murphy, S.E. Ong, F. Cervero-Liceras, N. Watt, D. Balabanova, S. Hogarth, W. Maimaris, K. Buse, M. McKee, P. Piot, and P. Perel, *Integrating cardiovascular diseases, hypertension, and diabetes with HIV services: a systematic review.* AIDS Care, 2018. **30**(1): p. 103-115.
18. Khabala, K.B., J.K. Edwards, B. Baruani, M. Sirengo, P. Musembi, R.J. Kosgei, K. Walter, J.M. Kibachio, M. Tondoi, H. Ritter, E. Wilkinson, and T. Reid, *Medication Adherence Clubs: a potential solution to managing large numbers of stable patients with multiple chronic diseases in informal settlements.* Trop Med Int Health, 2015. **20**(10): p. 1265-70.
19. Briggs, C.J., P. Capdegelle, and P. Garner, *Strategies for integrating primary health services in middle- and low-income countries: effects on performance, costs and patient outcomes.* Cochrane Database Syst Rev, 2001(4): p. CD003318.
20. Briggs, C.J. and P. Garner, *Strategies for integrating primary health services in middle- and low-income countries at the point of delivery.* Cochrane Database Syst Rev, 2006(2): p. CD003318.
21. Shayo, E., M.C. Van Hout, J. Birungi, A. Garrib, S. Kivuyo, S. Mfinanga, M.J. Nyrienda, I. Namakoola, J. Okebe, K. Ramaiya, M.O. Bachmann, W. Cullen, J.V. Lazarus, G. Gill, T. Shiri, D. Bukenya, H. Snell, M. Nanfuka, L.E. Cuevas, M. Shimwela, G. Mutungi, J. Musinguzi, J. Mghamba, K. Mugisha, S. Jaffar, P.G. Smith, and N.K. Sewankambo, *Ethical issues in intervention studies on the prevention and management of diabetes and hypertension in sub-Saharan Africa.* BMJ Glob Health, 2020. **5**(7).
22. Labhardt, N.D., J.R. Balo, M. Ndam, E. Manga, and B. Stoll, *Improved retention rates with low-cost interventions in hypertension and diabetes management in a rural African environment of nurse-led care: a cluster-randomised trial.* Trop Med Int Health, 2011. **16**(10): p. 1276-84.
23. Musicha, C., A.C. Crampin, N. Kayuni, O. Koole, A. Amberbir, B. Mwagomba, S. Jaffar, and M.J. Nyirenda, *Accessing clinical services and retention in care following*

For peer review only

screening for hypertension and diabetes among Malawian adults: an urban/rural comparison. J Hypertens, 2016. **34**(11): p. 2172-9.

24. Gill, G.V., C. Price, D. Shandu, M. Dedicoat, and D. Wilkinson, *An effective system of nurse-led diabetes care in rural Africa.* Diabet Med, 2008. **25**(5): p. 606-11.
25. Price, A.J., A.C. Crampin, A. Amberbir, N. Kayuni-Chihana, C. Musicha, T. Tafatatha, K. Branson, D.A. Lawlor, E. Mwaiyeghele, L. Nkhwazi, L. Smeeth, N. Pearce, E. Munthali, B.M. Mwagomba, C. Mwansambo, J.R. Glynn, S. Jaffar, and M. Nyirenda, *Prevalence of obesity, hypertension, and diabetes, and cascade of care in sub-Saharan Africa: a cross-sectional, population-based study in rural and urban Malawi.* Lancet Diabetes Endocrinol, 2018. **6**(3): p. 208-222.
26. Siedner, M.J., K. Baisley, J. Orne-Gliemann, D. Pillay, O. Koole, E.B. Wong, P. Matthews, F. Tanser, K. Herbst, T. Barnighausen, and M. Bachmann, *Linkage to primary care after home-based blood pressure screening in rural KwaZulu-Natal, South Africa: a population-based cohort study.* BMJ Open, 2018. **8**(12): p. e023369.
27. Rawat, A., K. Uebel, D. Moore, and A. Yassi, *Integrated HIV-Care Into Primary Health Care Clinics and the Influence on Diabetes and Hypertension Care: An Interrupted Time Series Analysis in Free State, South Africa Over 4 Years.* 2018. **77**(5): p. 476-483.
28. Matanje Mwagomba, B.L., S. Ameh, P. Bongomin, P.A. Juma, R.K. MacKenzie, C. Kyobutungi, N. Lukhele, K.J.M. Mwangi, A. Amberbir, K. Klipstein-Grobusch, F.X. Gomez-Olive, and J. Berman, *Opportunities and challenges for evidence-informed HIV-noncommunicable disease integrated care policies and programs: lessons from Malawi, South Africa, Swaziland and Kenya.* AIDS, 2018. **32 Suppl 1**: p. S21-S32.
29. Duffy, M., B. Ojikutu, S. Andrian, E. Sohng, T. Minior, and L.R. Hirschhorn, *Non-communicable diseases and HIV care and treatment: models of integrated service delivery.* Trop Med Int Health, 2017. **22**(8): p. 926-937.
30. Njuguna, B., S. Vorkoper, P. Patel, M.J.A. Reid, R. Vedanthan, C. Pfaff, P.H. Park, L. Fischer, J. Laktabai, and S.D. Pastakia, *Models of integration of HIV and noncommunicable disease care in sub-Saharan Africa: lessons learned and evidence gaps.* AIDS, 2018. **32 Suppl 1**: p. S33-S42.
31. Wroe, E.B., N. Kalanga, B. Mailosi, S. Mwalwanda, C. Kachimanga, K. Nyangulu, E. Dunbar, L. Kerr, L. Nazimera, and L. Dullie, *Leveraging HIV platforms to work toward comprehensive primary care in rural Malawi: the Integrated Chronic Care Clinic.* Healthc (Amst), 2015. **3**(4): p. 270-6.
32. Park, P.H. and S.D. Pastakia, *Access to Hemoglobin A1c in Rural Africa: A Difficult Reality with Severe Consequences.* J Diabetes Res, 2018. **2018**: p. 6093595.

For peer review only

Legends to Figures

Figure 1. Study schema

Figure 2. Kaplan-Meier curves showing probability proportion of patients of remaining in care according to condition.

For peer review only

Figure 1:

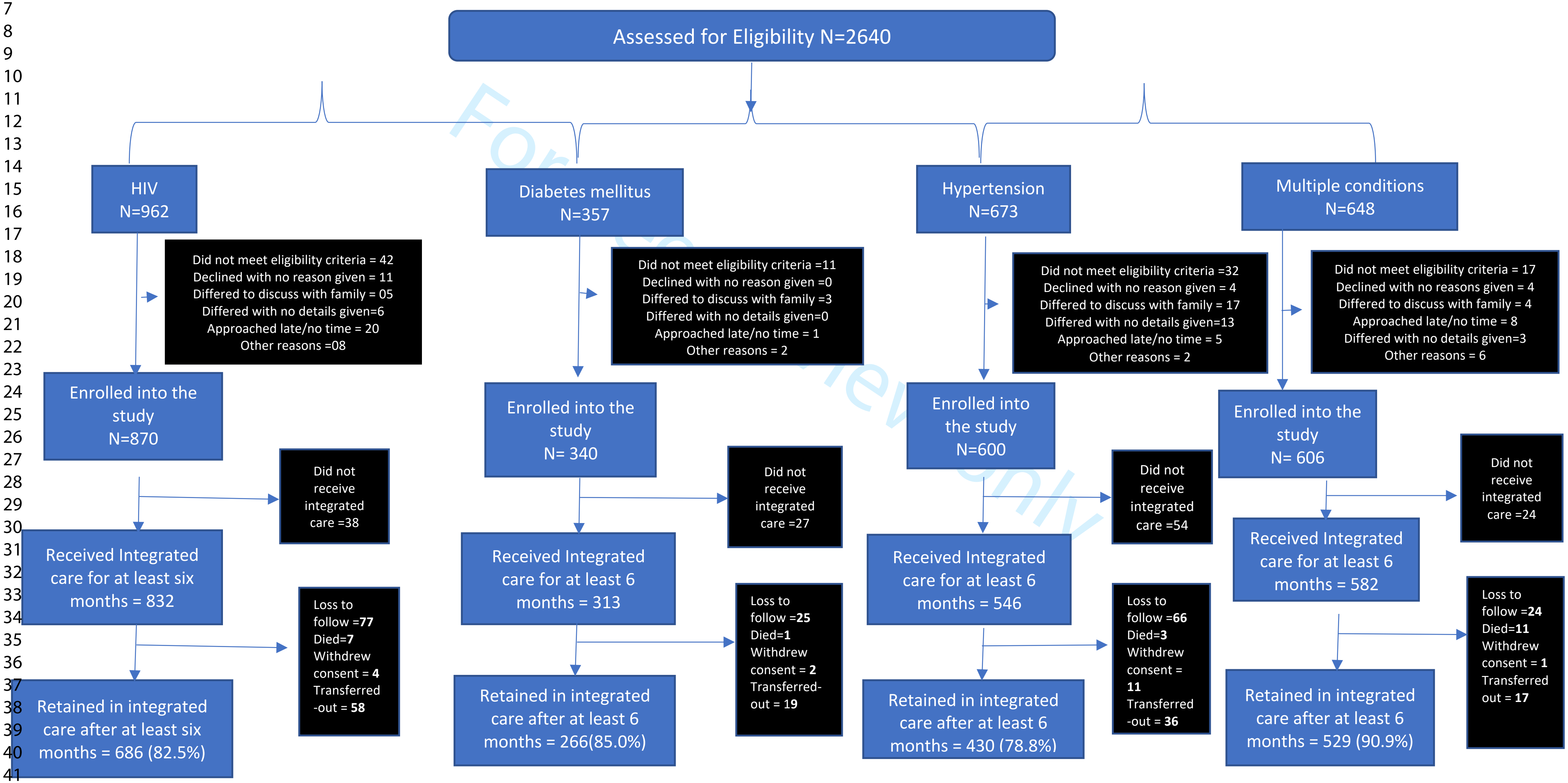

For peer review only - http://bmjopen.bmj.com/site/about/guidelines.xhtml

Figure 2.

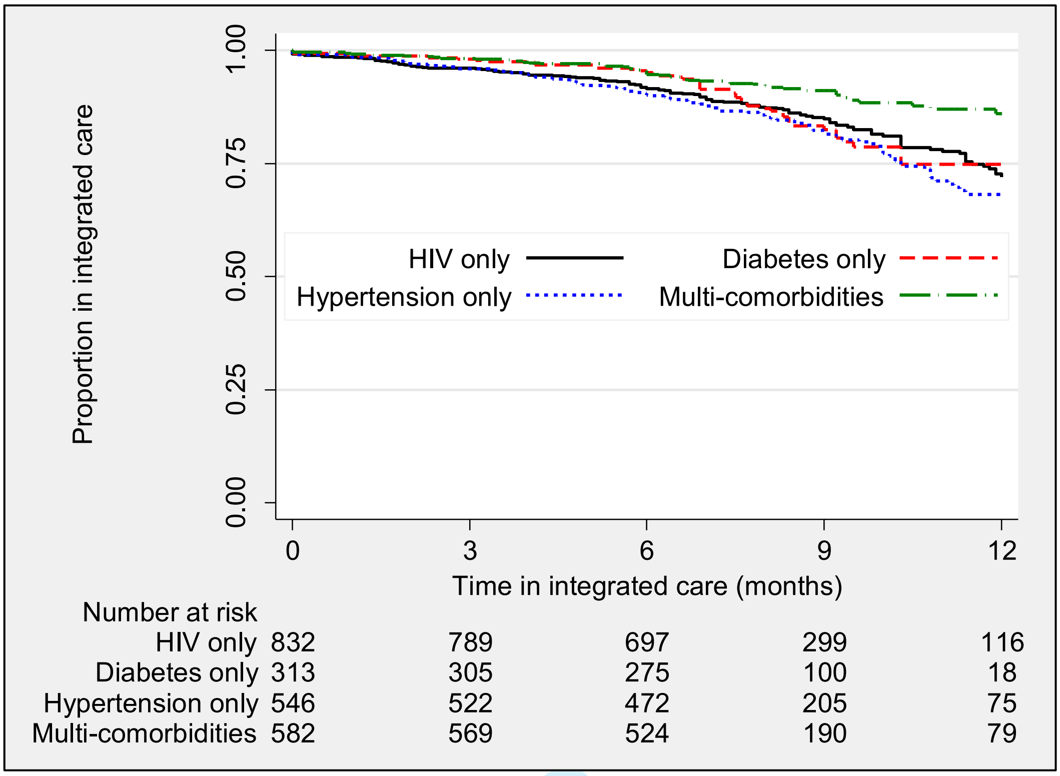

eview only

Appendix

Description of HIV, diabetes and hypertension services provided at MOCCA study facilities.

Country	Facility name	Facility type	Notes *
Tanzania	Amana	Regional Hospital	Separate dedicated clinics run for HIV, diabetes and hypertension
	Shree Hindu Mandal	Hospital (Non Governmental Organisation)	Separate dedicated clinics for HIV, diabetes and hypertension; partial integration already occurring.
	Mwananyamala	Regional Hospital	Separate dedicated clinics run for HIV, diabetes and hypertension
	Mkuranga	District Hospital	Dedicated HIV clinic. Outpatient management of diabetes and hypertension
	Bunju	Dispensary	Dedicated HIV-clinic. No clinic for diabetes or hypertension
Uganda	Kiswa	Health Centre, level III	Dedicated HIV clinic. Separate outpatient screening for diabetes and hypertension patients who are then referred for management to a higher facility as necessary.
	Kisugu	Health Centre, level III	Separate HIV clinic. Separate outpatient screening for diabetes and hypertension patients who are then referred for management to a higher facility as necessary.
	TASO Uganda	Non Governmental Organisation	Providing management and care services to patients living with HIV. Includes management of diabetes and hypertension for patients with HIV-infection.
	Ndejje	Health Centre, level IV	Separate dedicated clinics for HIV, diabetes and hypertension
	Wakiso	Health Centre, level IV	Separate dedicated clinics for HIV, diabetes and hypertension.

* Separate dedicated clinics involved separate waiting and consultation areas, a separate pharmacy, and separate appointments and medical records systems. The patients were seen by dedicated physicians.

For peer review only

BMJ Open

Integrating health services for HIV-infection, diabetes and hypertension in sub Saharan Africa: a cohort study.

Journal:	*BMJ Open*
Manuscript ID	bmjopen-2021-053412.R1
Article Type:	Original research
Date Submitted by the Author:	30-Aug-2021
Complete List of Authors:	Birungi, Josephine; MRC/UVRI and LSHTM Uganda Research Unit,<br>Kivuyo, Sokoine<br>Garrib, Anupam; Liverpool School of Tropical Medicine, ; Liverpool School of Tropical Medicine<br>Mugenyi, Levi; Infectious Disease Research Collaboration; I-Biostat, Hasselt University<br>Mutungi, Gerald<br>Namakoola, Ivan<br>Mghamba, Janneth<br>Ramaiya, Kaushik<br>Wang, Duolao; Liverpool School of Tropical Medicine<br>Maongezi, Sarah; Ministry of Health, Community Development, Gender, Elderly and Children, Dodoma, Tanzania<br>Musinguzi, Joshua<br>Mugisha , Kenneth<br>Etukoit, Bernard; The AIDS Support Organization<br>Kakande, Ayoub; MRC/UVRI and LSHTM Uganda Research Unit<br>Niessen, Louis; Liverpool School of Tropical Medicine<br>Okebe, Joseph<br>Shiri, Tinevimbo<br>Meshack, Shimwela<br>Lutale, Janet; Muhimbili University of Health and Allied Sciences<br>Gill, Geoff<br>Sewankambo, Nelson; Makerere University College of Health Sciences<br>Smith, Peter<br>Nyirenda, Moffat; Malawi Epidemiology and Intervention Research Unit (MEIRU); London School of Hygiene and Tropical Medicine Faculty of Epidemiology and Public Health<br>Mfinanga, Sayoki<br>Jaffar, Shabbar; Liverpool School of Tropical Medicine, Department of International Public Health
**Primary Subject Heading**:	Global health
Secondary Subject Heading:	Public health
Keywords:	Health policy < HEALTH SERVICES ADMINISTRATION & MANAGEMENT, Hypertension < CARDIOLOGY, DIABETES & ENDOCRINOLOGY, HIV & AIDS < INFECTIOUS DISEASES

SCHOLARONE™
Manuscripts

I, the Submitting Author has the right to grant and does grant on behalf of all authors of the Work (as defined in the below author licence), an exclusive licence and/or a non-exclusive licence for contributions from authors who are: i) UK Crown employees; ii) where BMJ has agreed a CC-BY licence shall apply, and/or iii) in accordance with the terms applicable for US Federal Government officers or employees acting as part of their official duties; on a worldwide, perpetual, irrevocable, royalty-free basis to BMJ Publishing Group Ltd ("BMJ") its licensees and where the relevant Journal is co-owned by BMJ to the co-owners of the Journal, to publish the Work in this journal and any other BMJ products and to exploit all rights, as set out in our licence.

The Submitting Author accepts and understands that any supply made under these terms is made by BMJ to the Submitting Author unless you are acting as an employee on behalf of your employer or a postgraduate student of an affiliated institution which is paying any applicable article publishing charge ("APC") for Open Access articles. Where the Submitting Author wishes to make the Work available on an Open Access basis (and intends to pay the relevant APC), the terms of reuse of such Open Access shall be governed by a Creative Commons licence – details of these licences and which Creative Commons licence will apply to this Work are set out in our licence referred to above.

Other than as permitted in any relevant BMJ Author's Self Archiving Policies, I confirm this Work has not been accepted for publication elsewhere, is not being considered for publication elsewhere and does not duplicate material already published. I confirm all authors consent to publication of this Work and authorise the granting of this licence.

For peer review only

Integrating health services for HIV-infection, diabetes and hypertension in sub-Saharan Africa: a cohort study.

CONFIDENTIAL – Please do not circulate

Josephine Birungi [1,2] *, Sokoine L Kivuyo[3] *, Anupam Garrib [4] *, Levicatus Mugenyi [1], Gerald Mutungi [5], Ivan Namakoola [2], Janneth Mghamba [6], Kaushik Ramaiya [7], Duolao Wang [4], Sarah Maongezi, Joshua Musinguzi [5], Kenneth Mugisha [1], Bernard Micheal Etukoit [1], Ayoub Kakande[2], Louis Niessen [4], Joseph Okebe [4], Tinevimbo Shiri [4], Meshack Shimwela [8], Janet Lutale [9], Geoff Gill [4], Nelson K Sewankambo [10], Peter G Smith [11], Moffat J Nyirenda [2, 12], Sayoki Mfinanga [3,4+], Shabbar Jaffar [4+]

* contributed equally (first authors)
\+ contributed equally (senior authors)

1. The AIDS Support Organisation, Mulago Hospital Complex, Kampala, Uganda
2. Medical Research Council/Uganda Virus Research Institute & London School of Hygiene and Tropical Medicine (MRC/UVRI & LSHTM), Uganda Research Unit
3. National Institutes for Medical Research, Dar es Salaam, Tanzania
4. Liverpool School of Tropical Medicine, Pembroke Place, Liverpool, L3 5QA
5. Non-Communicable Diseases and AIDS Control Programmes, Ministry of Health, Uganda
6. Directors Office, Ministry of Health, Community Development, Gender, Elderly and Children, Tanzania.
7. Hindu Mandal Hospital, Dar es Salaam, Tanzania
8. Amana Regional Referral Hospital, Dar es Salaam, Tanzania
9. Muhimbili University of Health and Allied Sciences, Dar es Salaam, Tanzania
10. Makerere University College of Health Sciences, Kampala, Uganda
11. MRC Tropical Epidemiology Group, London School of Hygiene & Tropical Medicine, Keppel Street, London WC1E 7HT
12. Medical Research Council / Uganda Virus Research Institute at the London School of Hygiene & Tropical Medicine Uganda Research Unit, Entebbe, Uganda.

Author for Correspondance: Prof Shabbar Jaffar, shabbar.jaffar@lstmed.ac.uk

For peer review only

Author contributions. . SJ, MJN, SMf, AG, GM, JMg, JMu, SMa, JL, GG, NKS and PGS designed the study with support from KR, KM, BE, LN. JB, SLK, IN implemented the study with support from AG, SMf, MJN, JO, TS, MS and KR. SLK and AK managed the data with support from JB and AG. JB, SLK, AG wrote the first drafts and SJ led the later drafts. LM did the statistical analysis with support from DW. JB co-ordinated the study in Uganda and SLK in Tanzania, with support from SJ, AG, MJN and SMf. All authors contributed to various drafts of the paper and saw and approved the final version.

Acknowledgements: We wish to thank Hazel Snell, Erik van Widenfelt, Luis Cuevas, the MOCCA study teams in Tanzania and Uganda and the health facility teams who supported the study and provided patient care, and Drs Mina Nakawuka Ssali and Martha Nabbada from the Ministry of Health in Uganda for their time and support, and Ayubu Masasi, Eric Mgina, Micheal Mubiru and Sebastian Owilla Kidega on data management.

Funding. This research was funded by the National Institute for Health Research (NIHR) (project reference 16/137/87) using UK aid from the UK Government to support global health research. The views expressed in this publication are those of the author(s) and not necessarily those of the NIHR or the UK Department of Health and Social Care.

Conflict of interest. The authors do not declare any conflict of interest

For peer review only

ABSTRACT

Background. HIV, diabetes and hypertension have a high disease burden in sub-Saharan Africa. Health care is organised in separate clinics, which may be inefficient. In a cohort study, we evaluated integrated management of these conditions from a single chronic care clinic.

Objectives: To determined the feasibility and acceptability of integrated management of chronic conditions in terms of retention in care and clinical indicators.

Design and Setting. Prospective cohort study comprising patients attending 10 health facilities offering primary care in Dar es Salaam and Kampala.

Intervention: Clinics within health facilities were set up to provide integrated care. Patients with either HIV, diabetes or hypertension had the same waiting areas, the same pharmacy, were seen by the same clinical staff, had similar provision of adherence counselling and tracking if they failed to attend appointments.

Primary outcome measures: retention in care, plasma viral load.

Strengths and limitations

- Study is multi-site and is largest ever conducted on integrating chronic care for HIV, diabetes and management in Africa.

- Study was done in close to normal health service conditions and so findings likely to be generalisable.

- The study was intended to determine feasibility and early clinical outcomes and does not have a comparison group.

BACKGROUND

The prevalence of diabetes and hypertension has risen sharply in Africa, where these conditions are now responsible for around two million premature adult deaths annually [1-6]. There are effective treatments for diabetes and hypertension, but only 10-20% of persons living with these conditions are thought to be in regular care[1, 7, 8], largely because service provision is limited [9-11]. Identifying approaches to increase access to health services for the management of diabetes and hypertension for populations in Africa represents one of the greatest public health challenges of our time.

Health services in most African countries are generally designed for dealing with acute infections and experience of chronic care management is limited [12], except for HIV-infection which, like other chronic diseases, requires life-long management. In Eastern and Southern Africa 83% of people who know they have HIV-infection are in regular care, with

For peer review only - http://bmjopen.bmj.com/site/about/guidelines.xhtml

90% of these virally suppressed[13], compared to a retention rate of about 50% a decade or so ago[14, 15]. HIV associated mortality has declined from over 2 million a year to less than half a million a year [13] and hypertension and diabetes will need to achieve similar levels of retention and disease control to achieve the large declines in mortality needed to meet the Sustainable Development Goals[16].

One reason why HIV services have been successful might be because they have been organised in stand-alone clinics in most African countries from shortly after the time that effective anti-retroviral drugs became available. People living with HIV-infection are managed in separate primary care clinics, with separate drug and diagnostic procurement channels and a separate funding stream. Now that the burden of other chronic conditions is a rapidly increasing public health problem, how should African health systems organise their services?

Managing multiple chronic conditions in the same clinics could reduce the duplication of health care resources and, crucially, could enable the learning acquired by HIV treatment programmes to be applied to the control of other chronic conditions. However, there is little evidence to support this in public health facilities [17, 18]. Integrated management could deter people with diabetes and hypertension in seeking care because of the stigma associated with HIV-infection. There is also a danger, that expanding the focus of health care provision in relatively weak health systems, could put at risk the gains achieved by HIV control programmes. In Tanzania and Uganda, working in close collaboration with policy makers and senior disease control managers, we conducted a cohort study of the integrated care for HIV-infection, diabetes and hypertension and evaluated its feasibility and acceptability in terms of retention in care and clinical indicators including control of blood pressure and fasting plasma glucose.

Methods

Study design and setting.

We conducted a prospective cohort study, known as the MOCCA study (Management of Chronic Conditions in Africa). We provided integrated care at 10 health facilities that were offering primary health care services, 5 in the Dar es Salaam region in Tanzania and 5 in and around Kampala, Uganda and followed up a cohort of participants over time to determine the feasibility and acceptability of this approach in terms of retention in care and clinical indicators. Appendix 1 describes the health facilities included.

The facilities were serving largely urban and peri-urban populations and chosen purposely. Eight were government run and 2 were run by non-government organisations. They ranged from regional hospitals through to smaller health centres and dispensaries [19, 20]. The larger facilities operated stand-alone separate clinics for diabetes and hypertension, while in the small facilities, patients with diabetes or hypertension were usually seen in the outpatient clinic. Each facility had a HIV clinic run separately from the rest of the clinical services. Patients attending HIV services had their own reception and waiting area, were seen by dedicated clinical staff, and had a separate pharmacy. Medical records, track and

For peer review only

trace procedures, and appointments also all differed from systems employed in other clinics in the facility.

What was integrated care? Integration involved the formation of a single clinic for the management of patients with HIV-infection, diabetes or hypertension or combinations of these conditions. All patients were seen by the same clinical staff. They shared a single reception, waiting areas and pharmacy. For patients with diabetes or hypertension, systems for tracking and follow up of patients, recording of clinical notes, counselling, appointments and registrations were mostly set up from new and aligned with those available for people living with HIV, such that all patients were managed in a similar way regardless of their condition.

How was integrated care implemented?

We had planned a brief observation period prior to implementing integrated care so to understand better the procedures at each clinic, such as the patient flow. We did this in the first 6 facilities. Very few patients had declined to join and thereafter we felt confident on how to set up the integrated care. Therefore, in the remaining 4 facilities, implementation was done straightaway without an observation period. In Uganda the first person received integrated care on the 5th October 2018 and recruitment finished on the 5th of April 2019. The final follow-up was on the 20th December 2019. In Tanzania, the first person received integrated care on 20th February 2019. Recruitment finished on 23rd June 2019. The final follow-up was on 30 January 2020.

We conducted assessments of each facility using a modified WHO Service Availability and Readiness Assessment (SARA) tool and reviewed routine data from the Health Management Information Systems (District Health Information System 2). This provided us with knowledge on the clinical infrastructure, availability of medicines, frequency and nature of patient attendance for assessments.

The integrated care clinic was set up to run separately as a stand-alone clinic at each facility so that patients who did not want integrated care would be able to continue with usual clinics. Organisation of the integrated care clinic, including the frequency of clinic days, was done by health facility managers. Where possible the integrated clinic was run on a different day from the HIV and hypertension or diabetes standalone clinics. In those facilities where this was not possible, it was run in a separate room away from the main standalone clinics. The integrated clinic was held initially on one day a week at all facilities and later increased to 2 days a week as the numbers of recruited participants increased. Clinical management was done by health care staff employed by the facilities and in accordance with local clinical practice and national guidelines.

The health care staff received refresher training in all three chronic health conditions to ensure a common level of understanding of clinical management. Certified national trainers conducted training in both countries. The training comprised a combination of two days of classroom sessions and on-the-job training. The doctors specialised in HIV and in non-communicable disease management conducted joint clinics for up to one month until they

For peer review only - http://bmjopen.bmj.com/site/about/guidelines.xhtml

felt comfortable to manage all three conditions. Classroom training included role play of different scenarios.

Counselling was generally not available for diabetes and hypertension management in either country prior to the study but was available routinely for patients with HIV-infection. We therefore provided training to nurses and counsellors on diabetes and hypertension, focussed on the need for high treatment adherence to prevent complications. It comprised one day of classroom training followed by on-the-job training for up to 4 weeks, as done for clinicians, and mentorship.

Prior to the study, HIV clinics had a system of tracking patients who did not attend a scheduled appointment. This generally involved contacting patients by phone if they missed an appointment, to determine the reason for the missed appointment and to reschedule. Such practices were not in place for people with either diabetes or hypertension. Therefore, we extended the system of tracking patients to cover all 3 conditions.

All health facility staff received training on the protocol and research methods. This was approximately one-day of classroom training, followed by on-the-job training.

Data collection: At each clinic, the research team employed one data clerk and one research nurse to inform patients about the research, collect research data and take informed consent. In addition, small amounts of salary (salary top-ups) were provided to selected facility staff working with study participants and to the head nurse responsible for the facility operations including overseeing the logistical set up of integrated care activities. Research staff worked alongside the health facility staff.

Selection of participants. In each disease-specific clinic a health talk was given to introduce the study. The number of patients with diabetes or with multiple conditions was very few. Therefore, all persons presenting with diabetes or with two or all 3 of our target conditions were invited to join the study. Both existing and newly diagnosed patients were enrolled. In the larger facilities (n=3, all in Tanzania), the number of people in care with HIV-infection or with hypertension was large and so we sampled systematically (usually every 20th patient) until the clinic ended for the day. In the remaining clinics, where patient load was lower and it was difficult to ascertain the order that patients had come to the facility, we enrolled patients who approached study staff to find out more, or clinicians approached patients and invited them to be screened for the study. On most clinic days, patients who had recently arrived at the facility came forward for enrolment, and those who had nearly completed their consultations for the day and were close to leaving the facility for home, were reluctant to come forward.

Provision of medicines for hypertension and diabetes

Before the study started, we recognised that supply of medicines for diabetes and hypertension was erratic in both countries. In Uganda, medicines for these conditions were normally provided free but shortages were common. In Tanzania, most patients were required to purchase medicines, and some had health insurance. We have discussed

For peer review only - http://bmjopen.bmj.com/site/about/guidelines.xhtml

elsewhere the ethical challenges of conducting research of this kind in situations of limited medicines supply[21].

To ensure a reliable supply of drugs, we purchased back-up supplies of basic drugs for diabetes and hypertension in both Tanzania and Uganda and supplemented patient supplies when the health service stock ran out. The medicines supplied were predominantly first line drugs and differed between countries depending on what was needed.

Due to the limited supply of medicines for diabetes and hypertension in Uganda, patients at one of the study facilities had organised themselves into a group that met and pooled funds on a monthly basis. These funds were used to purchase the medicines when the health facility ran out of stock. At this facility, we offered minimal buffer supplies compared to other facilities.

Measurements.

Retention was assessed through routine attendance at clinics. Patients who failed to attend an appointment were phoned to establish reasons for not attending and to reschedule. Three attempts were made to contact the patient by phone in the month following non-attendance at an appointment. A patient was regarded as lost to follow up if they had not attended an appointment in 6 months.

Blood pressure was measured using the Omron M6 comfort (OMRON Healthcare, Japan) in Uganda and in 3 of the facilities in Tanzania; Suresign SLD3-107 (CIGA Healthcare Ltd, UK) was used in the remaining facilities. We measured blood pressure at 3 time points in the sitting position, 5 minutes apart, and took the average of the three readings as the pressure. Hypertension was defined as a systolic pressure ≥140mmHg and/or a diastolic pressure ≥90mmHg and/or use of antihypertensive medication.

Plasma viral load testing was done by the Central Public Health laboratories in Tanzania and Uganda using the Cobas 8800 system (Roche). In Uganda, if the viral load result from the central laboratory was not available or was more than 6 months old, a test was done at the MRC/UVRI/LSHTM Uganda laboratory using Cobas Ampliprep. In both countries HbA1c testing was not routinely available in the public health system, and this was done by the study. In Uganda, HbA1c was tested by Cobas 6000 (Roche, Switzerland) at the MRC/UVRI/LSHTM Uganda laboratory, and in Tanzania at the Shree Hindu Mandal Hospital laboratory using a Siemens DCA Vantage (Siemens Healthcare Diagnostics, Germany). Fasting blood glucose was done using a point of care test in Tanzania (GlucoDr. Auto AGM-4000 and (all Medicus Co, Republic of Korea) and in Uganda Contour Plus (Bayer, Germany).

In Tanzania weight and height were measured using SECA RGZ-160 (SECA, GmbH & Co, Germany). In Uganda we used an ADE spring scale (kg, 1-150) for weight measurement and SECA 213 portable stadiometer (SECA, German) for height measurement. Weight and height were measured to the nearest ±0.1 kg and ±0.1 cm, respectively, and body mass index (BMI) was calculated as weight/height^2 (kg/m^2).

Patient and Public involvement

For peer review only

The concept of integrated care was developed in partnership with senior policy makers. In each country, we held planning meetings with the Ministry of Health and non-governmental partners at national and local level, with senior clinicians and hospital managers and with patient leaders and community representatives. National steering committees were formed, comprising representatives from these stakeholders together with the researchers and these committees contributed to the research strategy and guided its implementation. We also formed an international steering committee, that included 4 senior researchers and 4 independent researchers, which had oversight of the whole programme. We also held investigator meetings involving all of the partners and these included patient representatives and policy-makers.

Statistical analysis

The measure of retention used was defined as number in care at study end, calculated as the number enrolled into integrated care minus any deaths, losses-to-follow-up, transfers away from the health facility and withdrawals of consent.

Categorical variables were summarised using frequencies and proportions and compared using Chi-square tests. Continuous variables were summarised using means or medians and compared by a t-test or a Wilcoxon test, as appropriate. Generalized Estimating Equations (GEE) were used to account for clustering at the health facility level. Kaplan Meier curves and Cox regression with frailties (to adjust for clustering) were used to analyse retention times. The time was calculated as number of months from first attendance at the integrated clinic to the earliest of the end of the study, loss to follow up, death, withdrawal, or transfer-out. The analysis was done using STATA version 15.

Ethics clearance

The study received ethical clearance from TASO Research Ethics Committee and Uganda National Council for Science and Technology (UNCST) in Uganda (Reference TASO REC/015/18-UG-REC-009), National Health Research Ethics Review Sub-Committee in Tanzania (Reference NIMR/HQ/R.8a/Vol. IX/2793), and the Liverpool School of Tropical Medicine in the UK (Reference 18-044). No additional data are available.

RESULTS

Figure 1 shows the consort chart. Of 2640 patients assessed for eligibility, 102 (3.9%) did not meet eligibility criteria (34 were very sick, 50 were planning to move out of the area, 13 were pregnant and receiving care from antenatal care services, 5 other reasons), 19 (0.7%) declined and gave no reason, 51(1.9%) wanted more time to decide (mostly to discuss with their family), 34 (1.3%) were approached just as they had finished consultation and were leaving the clinic and 18 (0.7%) gave other reasons (mostly that they needed to be in specialist care).

Thus, of all those initially assessed for eligibility 91.5% (2416/2640) were invited to join integrated care. This invitation was given at their current clinics and they were asked to return at their next visit to the integrated care clinic. Six percent (143/2416) did not return

For peer review only

and thus 2,273/2640 (86.1%) were enrolled into integrated care, 927 in Tanzania and 1346 in Uganda.

Table 1 shows the characteristics of the participants at the start of integrated care, for each clinical condition. Overall, 74.5% (1689/2266) reported that they had been in care for 6-months or more prior to the start of the study. For people with multiple conditions, previous time in care was substantially greater than for those with single conditions. Overall, 71.1% (1615/2273) of those included in the study were female.

Median age varied substantially between the different conditions, with that for those living with HIV-infection being more than 10 years younger than participants in the other groups (p=0.0001, Kruskal Wallis test). Obesity was more common among participants with diabetes, hypertension or multimorbidity than with HIV-infection (504/1441 (35.0%) versus 116/832 (13.9%), risk ratio adjusted for clustering: 2.50, 95% CI: 1.88, 3.31, p<0.001). It was also substantially higher in women than in men (554/1615 (34.3%) vs 66/658 (10.0%), adjusted risk ratio: 3.40, 95% CI: 2.63– 4.41, p<0.001).

Table 2 shows the biomedical indicators as measured at baseline on the day of integration or within the previous 6 months. The majority did not reach target blood pressure and glycaemia control levels, whether they had a single condition or multimorbidity. When participants who reported that they had been in care for less than 6 months (i.e. patients who had newly started therapy) were excluded, 509/882 (57.7%) of those with hypertension had a blood pressure ≥140/90 mmHg, 355/508 (69.9%) of those with diabetes had a fasting blood glucose ≥7.0 mmol/L and 72/438 (16.4%) of those with HIV-infection had a plasma viral load ≥100 copies per ml.

The proportions alive and retained in care were high among all participants (Table 3). The overall proportion alive and in care among participants with a single condition was 1382/1691 (81.7%, 95% CI: 79.9% - 83.6%), which did not differ significantly from the proportion with multimorbidity (adjusted risk ratio 0.91, 95% CI: 0.82, 1.00, p=0.061).

Figure 2 shows the survival probability curves of being alive and in care over time. After adjusting for clustering, the probability (95% CI) of being retained and alive in care at 12 months was 0.74 (0.66 – 0.80) among participants with HIV alone, 0.74 (0.66– 0.81) among those with diabetes alone, 0.70 (0.64 – 0.76) among those with hypertension alone and 0.84 (0.79 – 0.88) among those with multimorbidity (p=0.008).

Table 4 shows the biomedical indicators of participants at the final follow-up. The proportions of patients living with HIV-infection with viral suppression were high, but the proportions of those with hypertension achieving blood pressure less than 140/90 mmHg and those with diabetes achieving glycaemia <7 mmol/L were substantially lower. However, blood pressure, fasting glucose and plasma viral load were all better controlled at study end than at baseline (Table 5), even when participants who had been in care for less than 6 months, and would have been newly started on therapy were excluded. After adjusting for clustering, among patients with HIV, the proportion (95% CI) with plasma viral load of <100 copies per ml was 88.5% (85.7%-91.4%) and those with plasma viral load <1000 copies per ml was 96.0% (94.3%-97.8%).

For peer review only - http://bmjopen.bmj.com/site/about/guidelines.xhtml

DISCUSSION

This study shows that integrating care for HIV-infection, diabetes and hypertension achieved high levels of retention in care for people living with diabetes or hypertension in Africa. Crucially, this improvement did not appear to be at the expense of HIV control, as retention in all 3 groups of people with single conditions was similar, and the proportion with an HIV viral suppression of less than a 100 copies per ml was close to 90%.

Thus, our study suggests that an integrated approach could achieve excellent retention for diabetes and hypertension in an integrated clinic, as has been achieved for people living with HIV-infection where retention exceeds 80% today, up from about 50% a decade or so ago[13-15].

The retention rate for people with diabetes or hypertension observed in our study was higher than that published from some other studies, which involved studies of single diseases, including those where diagnostic and treatment services were enhanced and, for example, treatments were provided for free[22-24]. In our study, we put in place basic measures to support participants with diabetes and hypertension including minimal access to basic medicines, counselling and telephone reminders for those who missed appointments. However, these measures were no more than those available to people in care with HIV-infection and no more than those available to people in research studies of hypertension and diabetes. No financial or other incentives were provided in our study and participants were managed by routine health care staff. Analysis of the costs of this package will be done separately but generic medicines for diabetes and hypertension and associated monitoring costs are substantially less than those for HIV-infection and so we anticipate that the model will be highly cost-effective.

In a large study in Malawi conducted by members of our team almost a decade ago, people identified with diabetes and hypertension in the community were referred into care with a dedicated medical officer and access to a free and reliable supply of medicines and diagnostics. However, at 12 months retention in care was 27% , suggesting that control of diabetes and hypertension when these conditions are identified through routine screening (i.e. are asymptomatic) might be a challenge[23, 25]. A study from Kwazulu-Natal in South Africa reported similar findings [26]. However, other studies that provided a good level of care and that have involved patients who know they have diabetes and hypertension, have also struggled to achieve good levels of retention[22].

Why did our study achieve high retention? For people with multiple conditions, a huge advantage of integrated care is that they only need to attend one clinic appointment, but in our study retention was also high for people with single conditions. It is possible that when services were integrated and the lessons learned in managing HIV-infection with respect to counselling, treatment adherence and the provision of a more streamlined service, were applied by health care staff to diabetes and hypertension management, patients responded to the changes in service delivery. The mixing of patients with HIV-infection with those living

For peer review only - http://bmjopen.bmj.com/site/about/guidelines.xhtml

with diabetes and hypertension may also have led to the sharing of experiences of living with a long-term condition, which could have brought further benefit.

We knew that integration of services would involve expansion of care responsibilities of health facility staff, and feared that this would adversely impact HIV care outcomes [17, 27, 28]. However, this did not happen. HIV viral suppression rates were high at enrolment and were slightly higher at the end of the study. There is an increasing body of literature on integrating screening, diagnosis and treatment for non-communicable diseases into established HIV services.[17, 29, 30] As far as we are aware, ours is the first study to test integration of services fully so that any person with one or more of the 3 target conditions could be managed in one clinic[29, 31].

We had feared that stigma may put off patients with diabetes and hypertension from attending a joint service with patients living with HIV. However, none of the patients who declined to join cited stigma. It is possible that the 41 patients who declined without giving a reason or who deferred their decision, did so because of the stigma. Nonetheless, these numbers of refusals are relatively small, and very few study participants withdrew over time. HIV has a special status in almost all African countries, which may have contributed to the stigma associated with this disease. Our study suggests that HIV-infection can be managed in the same space as other conditions – i.e. that HIV can be considered like any other disease or condition – and this will not make a difference to the outcome for the patient living with HIV-infection.

Although we achieved high rates of retention, and blood pressure and glycaemia control improved over time, a high percentage of those with diabetes or hypertension continued to have uncontrolled blood pressure and uncontrolled glycaemia at the end of the study, in contrast to the situation with HIV for which the vast majority maintained excellent control of viraemia. In the case of diabetes, this is complicated by a lack of access to adequate monitoring. Access to HbA1c testing was a challenge we faced in this study, and is a challenge across Africa[32]. The next step in this story is to identify and test interventions that improve blood pressure and diabetes control (among people in care) and take this control to levels achieved by HIV programmes in order to drive down mortality from non-communicable diseases.

Large numbers of people in high income countries are now living with multiple conditions – so called multimorbidity - and this number is expected to continue to rise sharply in Africa, if it has not done so. In our study, individuals with more than one condition, who would normally attend multiple different clinics, appeared to have a higher retention in care than people with a single condition but their clinical indicators at study end were no different from those with a single condition. While it is not surprising that their retention should be high, the lack of superiority in their clinical indicators from those with a single condition, suggest that greater efforts will be needed to improve clinical outcomes.

Studies that involve re-organisation of care provision represent huge change and challenges for health services, communities and patients. They are not possible without partnerships based on an ethos of equality and openness and without the building of trust between

For peer review only - http://bmjopen.bmj.com/site/about/guidelines.xhtml

researchers and these stakeholders. It is essential that research of this nature allows time and resource for the working together of different groups of researchers, health care providers and public health authorities, patients and communities.

Strengths and Limitations Although the evidence generated by our study is much greater than that available to most policy makers, and separate clinics for each chronic condition are not sustainable for Ministries of Health, changes to health policy that would affect tens of millions of people living with chronic conditions, requires robust evidence. Our study was large, comprising over 2000 participants with different chronic conditions. However, it was a pilot study and did not have a comparison group. It was done in just 10 health facilities, 5 in Tanzania and 5 in Uganda. In some health facilities, the number of people presenting with HIV or hypertension was high and the facilities had no system for recording the order in which patients presented and so we were unable to choose participants systematically but selected them purposively. This could have introduced selection bias.

Evidence is needed from large randomised trials with control groups comprising current standard care as provided in stand-alone clinics [17], with replication in multiple settings and over a longer duration of follow-up and with systematic or random sampling of participants to reduce the possibility bias. We also need more evidence on why integrated care works for different groups of people either with single or with multiple conditions.

For peer review only - http://bmjopen.bmj.com/site/about/guidelines.xhtml

Table 1. Baseline characteristics of patients studied.

**Number recruited into integrated care**		**HIV only**	**Diabetes only**	**Hypertension only**	**Multiple conditions**
	**Total**	**832**	**313**	**546**	**582**
	In Tanzania	352	164	160	251
	In Uganda	480	149	386	331
**Type of health facility**					
	Large public facilities	235	81	153	184
	Small public facilities	409	155	282	217
	NGO facilities	188	77	111	181
**Time in care prior to enrolment, number (%) ***					
	< 6 months	310 (37.4)	63 (20.3)	165 (30.3)	39 (6.7)
	6-11 months	41 (5.0)	28 (9.0)	42 (7.7)	22 (3.8)
	12-59 months	167 (20.1)	98 (31.6)	172 (31.6)	135 (23.2)
	>=60 months	311 (37.5)	121 (39.0)	166 (30.5)	386 (66.3)
**Median (range) months in care**		26.1 (0.0 – 343.4)	38.3 (0.0 – 431.6)	26.4 (0.0 – 566.2)	106 (0.0 – 534.8)
**Female, number (%)**		571 (68.6)	208 (66.5)	399 (73.1)	437 (75.1)
**Median age, (range)**		38 (18 – 78)	49 (19 – 77)	56 (27 – 86)	55 (21 – 85)
**BMI, number (%)**					
	<25	531 (63.8)	118 (37.7)	175 (32.1)	163 (28.0)
	25-29.9	185 (22.2)	98 (31.3)	181 (33.2)	202 (34.7)
	≥30	116 (13.9)	97 (31.0)	190 (34.8)	217 (37.3)
**Median BMI (range) in men**		21.1 (14.8 – 35.6)	24.3 (15.1 – 37.0)	25.1 (16.7 – 41.6)	26.0 (17.0 – 53.1)
**Median BMI (range) in women**		24.4 (13.2 – 48.9)	28.5 (15.4 – 48.2)	28.8 (16.0 – 65.2)	28.8 (15.8 – 57.9)

<u>**NOTES**</u>
* missing for 7 participants (3 with HIV-infection, 3 diabetes and 1 hypertension).

For peer review only

Table 2. Biomedical indicators at baseline.

	Patients who have a single condition occurring alone	The condition occurs with other conditions, causing multimorbidity (only among those with the condition)	Both combined
**AMONGST PATIENTS WITH HYPERTENSION**			
**Blood pressure (mmHg), number (%)**	**N=546**	**N=539**	**N=1085**
**≥140/90**	342 (62.6)	327 (60.7)	669 (61.7)
**<140/90**	204 (37.4)	212 (39.3)	416 (38.3)
**≥180/120**	42 (7.7)	64 (11.9)	106 (9.8)
**<180/120**	504 (92.3)	475 (88.1)	979 (90.2)
**Systolic BP mmHg, mean (range)**	144.4 (83.0 – 257.7)	145.1 (68.7 – 271.0)	144.7 (68.7 – 271.0)
**Diastolic BP mmHg, mean (range)**	90.2 (43.3 – 163.0)	90.3 (54.7 – 167.3)	90.2 (43.3 – 167.3)
**AMONGST PATIENTS WITH DIABETES**			
**Fasting glucose (mmol/L), number (%)**	**N=228**	**N=281**	**N=509**
<6.1	34 (14.9)	62 (22.1)	96 (18.9)
6.1–6.9	17 (7.5)	32 (11.4)	49 (9.6)
≥7.0	177 (77.6)	187 (66.6)	364 (71.5)
**Fasting glucose, mean (range) mmol/L**	11.5 (2.9 – 29.3)	9.8 (2.3 – 28.2)	10.5 (2.3 – 29.3)
**HbA1c, number (%) ***	**N=124**	**N=206**	**N=330**
(<6.0%	4 (3.2)	25 (12.1)	29 (8.8)
6.0–6.4%	7 (5.7)	22 (10.7)	29 (8.8)
≥6.5%	113 (91.1)	159 (77.2)	272 (82.4)
**HbA1c, mean (range) percent**	10.1 (4.8 – 16.9)	8.8 (4.8 – 16.5)	9.3 (4.8 – 16.9)
**AMONGST PATIENTS LIVING WITH HIV**			
**HIV plasma viral load, number (%) ***	**N= 493**	**N= 153**	**N= 646**
<100 copies/ml	306 (62.1)	131 (85.6)	437 (67.7)
100-999 copies/ml	68 (13.8)	14 (9.2)	82 (12.7)
≥1000 copies/ml	119 (24.1)	8 (5.2)	127 (19.7)

* measured at any time within the previous 6 months. Overall for the proportion with plasma viral load <100 copies/ml, the 95% CI was 64.1%, 71.4% and 80.3% (95% CI 77.3%-83.5%) had a plasma viral load < 1000 copies per ml.

Note: Blood pressure was measured at baseline by research staff among 1,187/1188 (99.9%) participants who were not known previously to be hypertensive. Of these, the proportion who were detected with a blood pressure of 140/90 mmHg or higher was 80/831 (9.6%) among those with HIV-infection alone, 47/313 (15.0%) among those with diabetes alone and 6/43 (14.0%) among those with HIV-infection and diabetes. Of note, among all HIV-infected participants, blood pressure was 180/120 mmHg or higher among 45/1126 (4.0%).

For peer review only

Table 3. Retention in integrated care and clinical outcomes according to condition.

**Overall**	**HIV only**	**Diabetes only**	**Hypertension only**	**Multiple conditions**
**Number enrolled into integration**	**832**	**313**	**546**	**582**
Median follow-up (inter-quartile range)	8.2 (6.9 – 10.4)	8.0 (6.9 – 9.4)	8.2 (6.9 – 10.1)	8.2 (7.2 – 9.8)
Retained in care to the end of the study n [% (95%CI)]*	n. 686 82.5 (79.9 –85.1)	n. 266 85.0 (81.1 – 89.0)	n. 430 78.8 (75.4 – 82.3)	n. 529 90.9 (88.6–93.3)
Rate per 100 person years (95% CI)*	75.0 (70.6, 78.7)	77.7 (70.4, 83.3)	69.9 (63.5, 74.6)	87.1 (83.2, 90.2)

* adjusted for clustering at health facility level

Notes: 85 persons were newly diagnosed with multiple conditions over the course of follow-up. Of participants with HIV alone, 5 were diagnosed with diabetes and 33 with hypertension. Of those with diabetes, 3 tested positive for HIV and 23 were diagnosed with hypertension. Of those with hypertension, 1 tested positive for HIV and 9 were diagnosed with diabetes. Eleven persons with dual conditions were diagnosed with a 3rd condition.

For peer review only

For peer review only - http://bmjopen.bmj.com/site/about/guidelines.xhtml

Table 4. Biomedical indicators at study end.

	Patients who have a single condition occurring alone	The condition occurs with other conditions, causing multimorbidity	Both combined
**Blood pressure (mmHg), number (%)**	**N=415**	**N=469**	**N=884**
≥140/90	183 (44.1)	225 (48.0)	408 (46.2)
<140/90	232 (55.9)	244 (51.2)	476 (53.9)
≥180/120	7 (1.7)	25 (5.3)	32 (3.6)
<180/120	408 (98.3)	444 (94.7)	852 (96.4)
**Systolic BP mmHg, mean (range)**	133.9 (26.0 – 228)	136.7 (90.0 – 227)	135.4 (26 – 228)
**Diastolic BP mmHg, mean (range)**	83.9 (53 – 130)	85.6 (44 – 185)	84.8 (44 – 185)
**Fasting glucose (mmol/L), number (%)**	**N=189**	**N=242**	**N=431**
<6.1	36 (19.1)	69 (28.5)	105 (24.4)
6.1–6.9	28 (14.8)	33 (13.6)	61 (14.2)
≥7.0	125 (66.1)	140 (57.9)	265 (61.5)
**Fasting glucose, mean (range) mmol/L**	9.7 (3.6 – 28.5)	8.3 (3.6 – 19.8)	8.9 (3.6 – 28.5)
**HbA1c, number (%).**	**N=84**	**N=140**	**N=224**
(<6%	17 (20.2)	31 (22.1)	48 (21.4)
6.0-6.4%	4 (4.8)	15 (10.7)	19 (8.5)
≥6.5%	63 (75.0)	94 (67.1)	157 (70.1)
**HbA1c, mean (range) percent**	7.6 (0.6 – 13.5)	7.3 (0.6 – 15.9)	7.4 (0.6 – 15.9)
**HIV plasma viral load, number (%) ***	**N=322**	**N=156**	**N=478**
<100 copies/ml	278 (86.3)	145 (93.0)	423 (88.5)
100-999 copies/ml	27 (8.4)	9 (5.8)	36 (7.5)
>=1000 copies/ml	17 (5.3)	2 (1.3)	19 (4.0)

* The 95% CI for plasma viral load<100 copies/ml for both groups combined was 85.7, 91.4%). The proportion with plasma viral load<1000 copies per ml was 96.0% (95% CI 94.3%, 97.8%).

Note: 80 persons were newly diagnosed with 2nd or 3rd conditions over the course of the follow-up (see Table 3). Their final follow-up indicators are not included in the analyses above as these patients may have been newly started on treatment and therefore will not have had time to stabilise.

For peer review only

Table 5. Risk differences (95% CI) between baseline and study end among participants living with hypertension, diabetes or HIV-infection.

Clinical condition (either alone or alongside other conditions)	All participants	Excluding participants in care for less 6 months at enrolment
Raised blood pressure (≥140/90 mmHg)	14.8%, (10.5%, 19.2%) (p<0.001)	10.5%, (5.7%, 15.3%) p<0.001).
Raised fasting glucose (≥7 mmol/L)	8.4%, (2.4%, 14.3%) p=0.006)	7.4% (1.0%, 13.7%) p=0.024)
Lack of viral suppression (plasma viral load ≥ 100 copies per ml)	20.8%, (16.0%, 25.5%) p<0.001).	7.3% (2.7%, 11.9%) p=0.002)

Notes: the risk difference is calculated as risk estimate at baseline minus risk estimate at study end.

For peer review only

References

1. Atun, R., J.I. Davies, E.A.M. Gale, T. Barnighausen, D. Beran, A.P. Kengne, N.S. Levitt, F.W. Mangugu, M.J. Nyirenda, G.D. Ogle, K. Ramaiya, N.K. Sewankambo, E. Sobngwi, S. Tesfaye, J.S. Yudkin, S. Basu, C. Bommer, E. Heesemann, J. Manne-Goehler, I. Postolovska, V. Sagalova, S. Vollmer, Z.G. Abbas, B. Ammon, M.T. Angamo, A. Annamreddi, A. Awasthi, S. Besancon, S. Bhadriraju, A. Binagwaho, P.I. Burgess, M.J. Burton, J. Chai, F.P. Chilunga, P. Chipendo, A. Conn, D.R. Joel, A.W. Eagan, C. Gishoma, J. Ho, S. Jong, S.S. Kakarmath, Y. Khan, R. Kharel, M.A. Kyle, S.C. Lee, A. Lichtman, C.P. Malm, M.N. Mbaye, M.A. Muhimpundu, B.M. Mwagomba, K.J. Mwangi, M. Nair, S.P. Niyonsenga, B. Njuguna, O.L.O. Okafor, O. Okunade, P.H. Park, S.D. Pastakia, C. Pekny, A. Reja, C.N. Rotimi, S. Rwunganira, D. Sando, G. Sarriera, A. Sharma, A. Sidibe, E.S. Siraj, A.S. Syed, K. Van Acker, and M. Werfalli, *Diabetes in sub-Saharan Africa: from clinical care to health policy.* Lancet Diabetes Endocrinol, 2017. **5**(8): p. 622-667.
2. Bosu, W.K., S.T. Reilly, J.M.K. Aheto, and E. Zucchelli, *Hypertension in older adults in Africa: A systematic review and meta-analysis.* PLoS One, 2019. **14**(4): p. e0214934.
3. Guwatudde, D., J. Nankya-Mutyoba, R. Kalyesubula, C. Laurence, C. Adebamowo, I. Ajayi, F. Bajunirwe, M. Njelekela, F.S. Chiwanga, T. Reid, J. Volmink, H.O. Adami, M.D. Holmes, and S. Dalal, *The burden of hypertension in sub-Saharan Africa: a four-country cross sectional study.* BMC Public Health, 2015. **15**: p. 1211.
4. Mills, K.T., J.D. Bundy, T.N. Kelly, J.E. Reed, P.M. Kearney, K. Reynolds, J. Chen, and J. He, *Global Disparities of Hypertension Prevalence and Control: A Systematic Analysis of Population-Based Studies From 90 Countries.* Circulation, 2016. **134**(6): p. 441-50.
5. Organisation, W.H., *Descrption status report on non-communicable diseases 2010.* 2011, 2010(Geneva, Switzerland).
6. Sarki, A.M., C.U. Nduka, S. Stranges, N.B. Kandala, and O.A. Uthman, *Prevalence of Hypertension in Low- and Middle-Income Countries: A Systematic Review and Meta-Analysis.* Medicine (Baltimore), 2015. **94**(50): p. e1959.
7. Addo, J., L. Smeeth, and D.A. Leon, *Hypertension in sub-saharan Africa: a systematic review.* Hypertension, 2007. **50**(6): p. 1012-8.
8. Geldsetzer, P., J. Manne-Goehler, M.E. Marcus, C. Ebert, Z. Zhumadilov, C.S. Wesseh, L. Tsabedze, A. Supiyev, L. Sturua, S.K. Bahendeka, A.M. Sibai, S. Quesnel-Crooks, B. Norov, K.J. Mwangi, O. Mwalim, R. Wong-McClure, M.T. Mayige, J.S. Martins, N. Lunet, D. Labadarios, K.B. Karki, G.B. Kagaruki, J.M.A. Jorgensen, N.C. Hwalla, D. Houinato, C. Houehanou, M. Msaidie, D. Guwatudde, M.S. Gurung, G. Gathecha, M. Dorobantu, A. Damasceno, P. Bovet, B.W. Bicaba, K.K. Aryal, G. Andall-Brereton, K. Agoudavi, A. Stokes, J.I. Davies, T. Barnighausen, R. Atun, S. Vollmer, and L.M. Jaacks, *The state of hypertension care in 44 low-income and middle-income countries: a cross-sectional study of nationally representative individual-level data from 1.1 million adults.* Lancet, 2019. **394**(10199): p. 652-662.
9. Katende, D., G. Mutungi, K. Baisley, S. Biraro, E. Ikoona, R. Peck, L. Smeeth, R. Hayes, P. Munderi, and H. Grosskurth, *Readiness of Ugandan health services for the management of outpatients with chronic diseases.* Trop Med Int Health, 2015. **20**(10): p. 1385-95.
10. Peck, R., J. Mghamba, F. Vanobberghen, B. Kavishe, V. Rugarabamu, L. Smeeth, R. Hayes, H. Grosskurth, and S. Kapiga, *Preparedness of Tanzanian health facilities for*

For peer review only - http://bmjopen.bmj.com/site/about/guidelines.xhtml

outpatient primary care of hypertension and diabetes: a cross-sectional survey. Lancet Glob Health, 2014. **2**(5): p. e285-92.

11. Manne-Goehler, J., R. Atun, A. Stokes, A. Goehler, D. Houinato, C. Houehanou, M.M. Hambou, B.L. Mbenza, E. Sobngwi, N. Balde, J.K. Mwangi, G. Gathecha, P.W. Ngugi, C.S. Wesseh, A. Damasceno, N. Lunet, P. Bovet, D. Labadarios, K. Zuma, M. Mayige, G. Kagaruki, K. Ramaiya, K. Agoudavi, D. Guwatudde, S.K. Bahendeka, G. Mutungi, P. Geldsetzer, N.S. Levitt, J.A. Salomon, J.S. Yudkin, S. Vollmer, and T. Barnighausen, *Diabetes diagnosis and care in sub-Saharan Africa: pooled analysis of individual data from 12 countries.* Lancet Diabetes Endocrinol, 2016. **4**(11): p. 903-912.
12. Garrib, A., J. Birungi, S. Lesikari, I. Namakoola, T. Njim, L. Cuevas, L. Niessen, K. Mugisha, G. Mutungi, J. Mghamba, K. Ramaiya, S. Jaffar, S. Mfinanga, and M. Nyirenda, *Integrated care for human immunodeficiency virus, diabetes and hypertension in Africa.* Trans R Soc Trop Med Hyg, 2019. **113**(12): p. 809-812.
13. UNAIDS, *UNAIDS Data 2020*. 2020, UNAIDS: Geneva, Switzerland.
14. Rosen, S., M.P. Fox, and C.J. Gill, *Patient retention in antiretroviral therapy programs in sub-Saharan Africa: a systematic review.* PLoS Med, 2007. **4**(10): p. e298.
15. Rosen, S. and M.P. Fox, *Retention in HIV care between testing and treatment in sub-Saharan Africa: a systematic review.* PLoS Med, 2011. **8**(7): p. e1001056.
16. collaborators, N.C.D.C., *NCD Countdown 2030: pathways to achieving Sustainable Development Goal target 3.4.* Lancet, 2020. **396**(10255): p. 918-934.
17. Haldane, V., H. Legido-Quigley, F.L.H. Chuah, L. Sigfrid, G. Murphy, S.E. Ong, F. Cervero-Liceras, N. Watt, D. Balabanova, S. Hogarth, W. Maimaris, K. Buse, M. McKee, P. Piot, and P. Perel, *Integrating cardiovascular diseases, hypertension, and diabetes with HIV services: a systematic review.* AIDS Care, 2018. **30**(1): p. 103-115.
18. Khabala, K.B., J.K. Edwards, B. Baruani, M. Sirengo, P. Musembi, R.J. Kosgei, K. Walter, J.M. Kibachio, M. Tondoi, H. Ritter, E. Wilkinson, and T. Reid, *Medication Adherence Clubs: a potential solution to managing large numbers of stable patients with multiple chronic diseases in informal settlements.* Trop Med Int Health, 2015. **20**(10): p. 1265-70.
19. Briggs, C.J., P. Capdegelle, and P. Garner, *Strategies for integrating primary health services in middle- and low-income countries: effects on performance, costs and patient outcomes.* Cochrane Database Syst Rev, 2001(4): p. CD003318.
20. Briggs, C.J. and P. Garner, *Strategies for integrating primary health services in middle- and low-income countries at the point of delivery.* Cochrane Database Syst Rev, 2006(2): p. CD003318.
21. Shayo, E., M.C. Van Hout, J. Birungi, A. Garrib, S. Kivuyo, S. Mfinanga, M.J. Nyrienda, I. Namakoola, J. Okebe, K. Ramaiya, M.O. Bachmann, W. Cullen, J.V. Lazarus, G. Gill, T. Shiri, D. Bukenya, H. Snell, M. Nanfuka, L.E. Cuevas, M. Shimwela, G. Mutungi, J. Musinguzi, J. Mghamba, K. Mugisha, S. Jaffar, P.G. Smith, and N.K. Sewankambo, *Ethical issues in intervention studies on the prevention and management of diabetes and hypertension in sub-Saharan Africa.* BMJ Glob Health, 2020. **5**(7).
22. Labhardt, N.D., J.R. Balo, M. Ndam, E. Manga, and B. Stoll, *Improved retention rates with low-cost interventions in hypertension and diabetes management in a rural African environment of nurse-led care: a cluster-randomised trial.* Trop Med Int Health, 2011. **16**(10): p. 1276-84.
23. Musicha, C., A.C. Crampin, N. Kayuni, O. Koole, A. Amberbir, B. Mwagomba, S. Jaffar, and M.J. Nyirenda, *Accessing clinical services and retention in care following*

For peer review only

screening for hypertension and diabetes among Malawian adults: an urban/rural comparison. J Hypertens, 2016. **34**(11): p. 2172-9.

24. Gill, G.V., C. Price, D. Shandu, M. Dedicoat, and D. Wilkinson, *An effective system of nurse-led diabetes care in rural Africa.* Diabet Med, 2008. **25**(5): p. 606-11.
25. Price, A.J., A.C. Crampin, A. Amberbir, N. Kayuni-Chihana, C. Musicha, T. Tafatatha, K. Branson, D.A. Lawlor, E. Mwaiyeghele, L. Nkhwazi, L. Smeeth, N. Pearce, E. Munthali, B.M. Mwagomba, C. Mwansambo, J.R. Glynn, S. Jaffar, and M. Nyirenda, *Prevalence of obesity, hypertension, and diabetes, and cascade of care in sub-Saharan Africa: a cross-sectional, population-based study in rural and urban Malawi.* Lancet Diabetes Endocrinol, 2018. **6**(3): p. 208-222.
26. Siedner, M.J., K. Baisley, J. Orne-Gliemann, D. Pillay, O. Koole, E.B. Wong, P. Matthews, F. Tanser, K. Herbst, T. Barnighausen, and M. Bachmann, *Linkage to primary care after home-based blood pressure screening in rural KwaZulu-Natal, South Africa: a population-based cohort study.* BMJ Open, 2018. **8**(12): p. e023369.
27. Rawat, A., K. Uebel, D. Moore, and A. Yassi, *Integrated HIV-Care Into Primary Health Care Clinics and the Influence on Diabetes and Hypertension Care: An Interrupted Time Series Analysis in Free State, South Africa Over 4 Years.* 2018. **77**(5): p. 476-483.
28. Matanje Mwagomba, B.L., S. Ameh, P. Bongomin, P.A. Juma, R.K. MacKenzie, C. Kyobutungi, N. Lukhele, K.J.M. Mwangi, A. Amberbir, K. Klipstein-Grobusch, F.X. Gomez-Olive, and J. Berman, *Opportunities and challenges for evidence-informed HIV-noncommunicable disease integrated care policies and programs: lessons from Malawi, South Africa, Swaziland and Kenya.* AIDS, 2018. **32 Suppl 1**: p. S21-S32.
29. Duffy, M., B. Ojikutu, S. Andrian, E. Sohng, T. Minior, and L.R. Hirschhorn, *Non-communicable diseases and HIV care and treatment: models of integrated service delivery.* Trop Med Int Health, 2017. **22**(8): p. 926-937.
30. Njuguna, B., S. Vorkoper, P. Patel, M.J.A. Reid, R. Vedanthan, C. Pfaff, P.H. Park, L. Fischer, J. Laktabai, and S.D. Pastakia, *Models of integration of HIV and noncommunicable disease care in sub-Saharan Africa: lessons learned and evidence gaps.* AIDS, 2018. **32 Suppl 1**: p. S33-S42.
31. Wroe, E.B., N. Kalanga, B. Mailosi, S. Mwalwanda, C. Kachimanga, K. Nyangulu, E. Dunbar, L. Kerr, L. Nazimera, and L. Dullie, *Leveraging HIV platforms to work toward comprehensive primary care in rural Malawi: the Integrated Chronic Care Clinic.* Healthc (Amst), 2015. **3**(4): p. 270-6.
32. Park, P.H. and S.D. Pastakia, *Access to Hemoglobin A1c in Rural Africa: A Difficult Reality with Severe Consequences.* J Diabetes Res, 2018. **2018**: p. 6093595.

For peer review only

Legends to Figures

Figure 1. Study schema

Figure 2. Kaplan-Meier curves showing probability proportion of patients of remaining in care according to condition.

For peer review only

For peer review only - http://bmjopen.bmj.com/site/about/guidelines.xhtml

Figure 1:

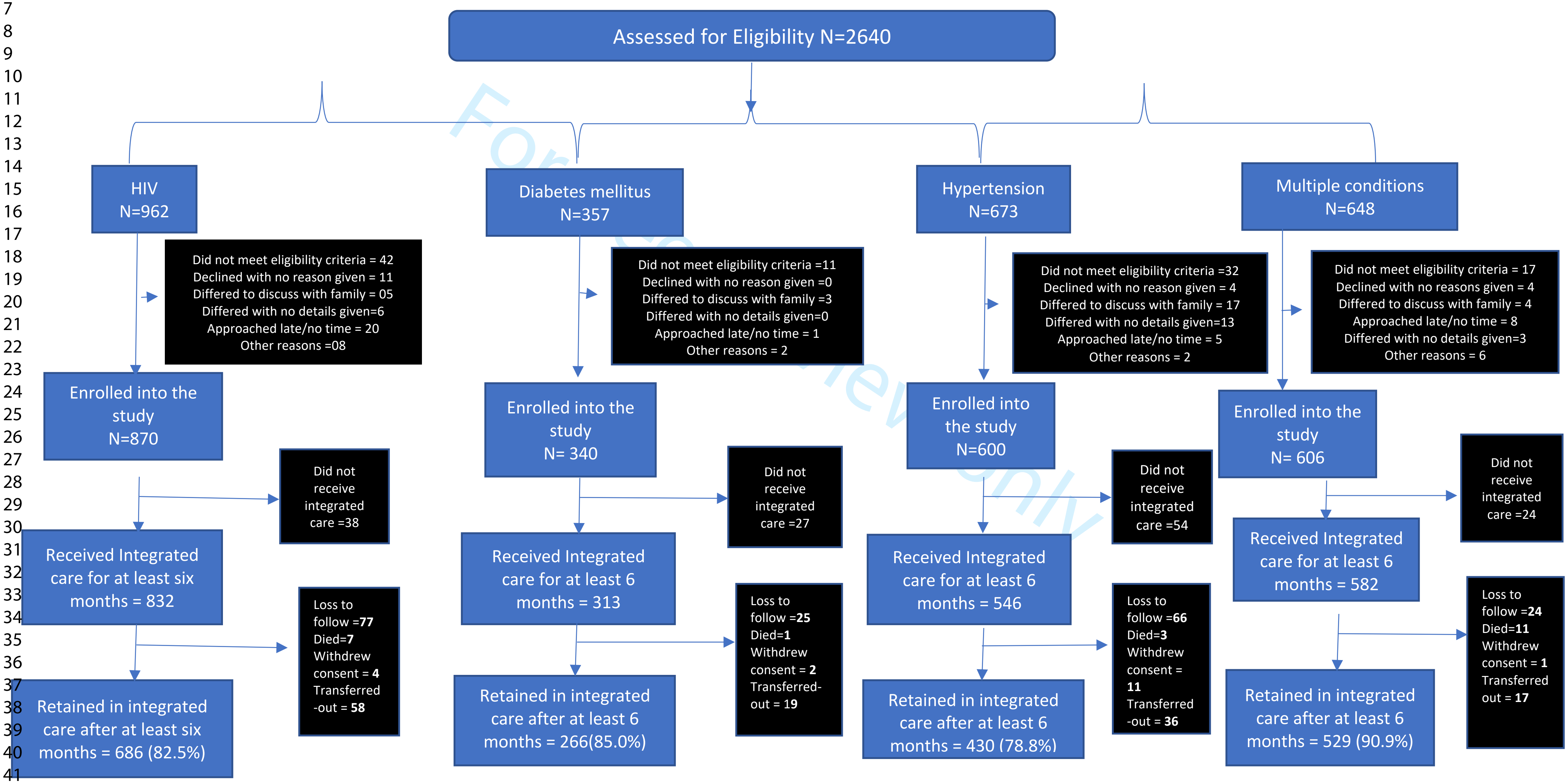

For peer review only - http://bmjopen.bmj.com/site/about/guidelines.xhtml

Figure 2.

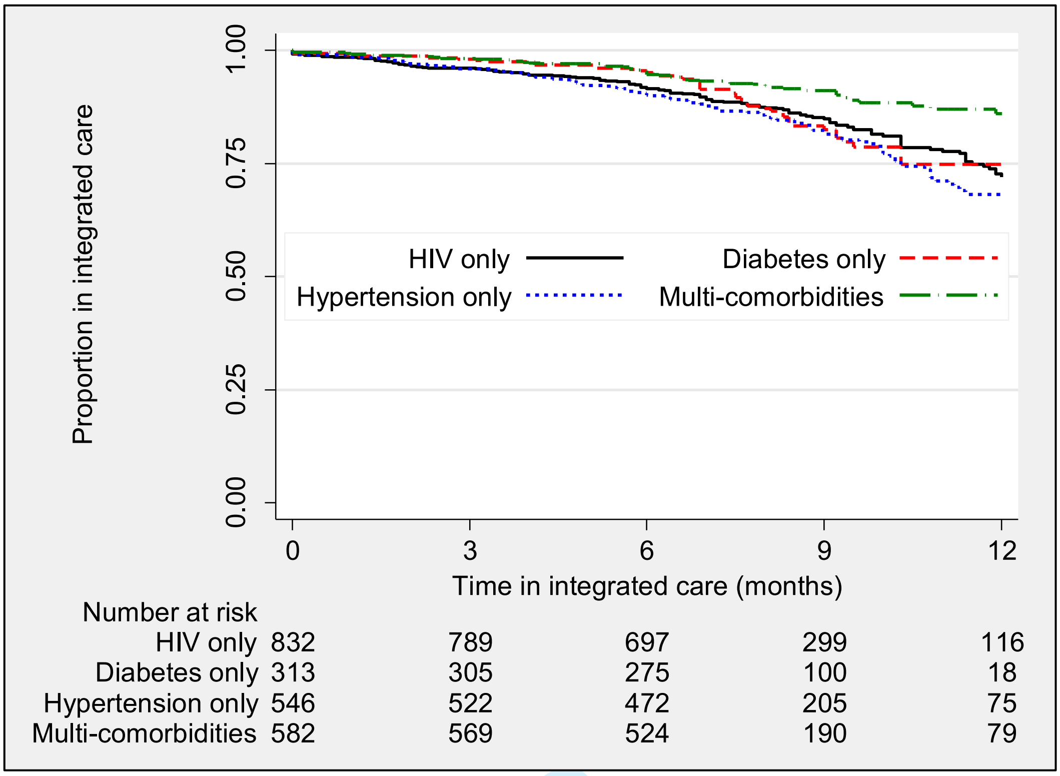

review only

Appendix

Description of HIV, diabetes and hypertension services provided at MOCCA study facilities.

Country	Facility name	Facility type	Notes *
Tanzania	Amana	Regional Hospital	Separate dedicated clinics run for HIV, diabetes and hypertension
	Shree Hindu Mandal	Hospital (Non Governmental Organisation)	Separate dedicated clinics for HIV, diabetes and hypertension; partial integration already occurring.
	Mwananyamala	Regional Hospital	Separate dedicated clinics run for HIV, diabetes and hypertension
	Mkuranga	District Hospital	Dedicated HIV clinic. Outpatient management of diabetes and hypertension
	Bunju	Dispensary	Dedicated HIV-clinic. No clinic for diabetes or hypertension
Uganda	Kiswa	Health Centre, level III	Dedicated HIV clinic. Separate outpatient screening for diabetes and hypertension patients who are then referred for management to a higher facility as necessary.
	Kisugu	Health Centre, level III	Separate HIV clinic. Separate outpatient screening for diabetes and hypertension patients who are then referred for management to a higher facility as necessary.
	TASO Uganda	Non Governmental Organisation	Providing management and care services to patients living with HIV. Includes management of diabetes and hypertension for patients with HIV-infection.
	Ndejje	Health Centre, level IV	Separate dedicated clinics for HIV, diabetes and hypertension
	Wakiso	Health Centre, level IV	Separate dedicated clinics for HIV, diabetes and hypertension.

* Separate dedicated clinics involved separate waiting and consultation areas, a separate pharmacy, and separate appointments and medical records systems. The patients were seen by dedicated physicians.

For peer review only

BMJ Open

Integrating health services for HIV-infection, diabetes and hypertension in sub Saharan Africa: a cohort study.

Journal:	*BMJ Open*
Manuscript ID	bmjopen-2021-053412.R2
Article Type:	Original research
Date Submitted by the Author:	13-Oct-2021
Complete List of Authors:	Birungi, Josephine; MRC/UVRI and LSHTM Uganda Research Unit,<br>Kivuyo, Sokoine; National Institute for Medical Research Muhimbili Research Centre<br>Garrib, Anupam; Liverpool School of Tropical Medicine, ; Liverpool School of Tropical Medicine<br>Mugenyi, Levi; Infectious Disease Research Collaboration; I-Biostat, Hasselt University<br>Mutungi, Gerald; Minstry of Health<br>Namakoola, Ivan; MRC/UVRI and LSHTM Uganda Research Unit<br>Mghamba, Janneth; Minstry of Health<br>Ramaiya, Kaushik; Shree Hindu Mandal Hospital<br>Wang, Duolao; Liverpool School of Tropical Medicine<br>Maongezi, Sarah; Ministry of Health, Community Development, Gender, Elderly and Children, Dodoma, Tanzania<br>Musinguzi, Joshua; Ministry of Health<br>Mugisha , Kenneth; The AIDS Support Organization<br>Etukoit, Bernard; The AIDS Support Organization<br>Kakande, Ayoub; MRC/UVRI and LSHTM Uganda Research Unit<br>Niessen, Louis; Liverpool School of Tropical Medicine<br>Okebe, Joseph; Liverpool School of Tropical Medicine<br>Shiri, Tinevimbo; Liverpool School of Tropical Medicine<br>Meshack, Shimwela ; Minstry of Health<br>Lutale, Janet; Muhimbili University of Health and Allied Sciences<br>Gill, Geoff; Liverpool School of Tropical Medicine<br>Sewankambo, Nelson; Makerere University College of Health Sciences<br>Smith, Peter; London School of Hygiene & Tropical Medicine<br>Nyirenda, Moffat; Malawi Epidemiology and Intervention Research Unit (MEIRU); London School of Hygiene and Tropical Medicine Faculty of Epidemiology and Public Health<br>Mfinanga, Sayoki; NIMR<br>Jaffar, Shabbar; Liverpool School of Tropical Medicine, Department of International Public Health
**Primary Subject Heading**:	Global health
Secondary Subject Heading:	Public health
Keywords:	Health policy < HEALTH SERVICES ADMINISTRATION & MANAGEMENT, Hypertension < CARDIOLOGY, DIABETES & ENDOCRINOLOGY, HIV &

	AIDS < INFECTIOUS DISEASES

SCHOLARONE™
Manuscripts

I, the Submitting Author has the right to grant and does grant on behalf of all authors of the Work (as defined in the below author licence), an exclusive licence and/or a non-exclusive licence for contributions from authors who are: i) UK Crown employees; ii) where BMJ has agreed a CC-BY licence shall apply, and/or iii) in accordance with the terms applicable for US Federal Government officers or employees acting as part of their official duties; on a worldwide, perpetual, irrevocable, royalty-free basis to BMJ Publishing Group Ltd ("BMJ") its licensees and where the relevant Journal is co-owned by BMJ to the co-owners of the Journal, to publish the Work in this journal and any other BMJ products and to exploit all rights, as set out in our licence.

The Submitting Author accepts and understands that any supply made under these terms is made by BMJ to the Submitting Author unless you are acting as an employee on behalf of your employer or a postgraduate student of an affiliated institution which is paying any applicable article publishing charge ("APC") for Open Access articles. Where the Submitting Author wishes to make the Work available on an Open Access basis (and intends to pay the relevant APC), the terms of reuse of such Open Access shall be governed by a Creative Commons licence – details of these licences and which Creative Commons licence will apply to this Work are set out in our licence referred to above.

Other than as permitted in any relevant BMJ Author's Self Archiving Policies, I confirm this Work has not been accepted for publication elsewhere, is not being considered for publication elsewhere and does not duplicate material already published. I confirm all authors consent to publication of this Work and authorise the granting of this licence.

For peer review only

Integrating health services for HIV-infection, diabetes and hypertension in sub-Saharan Africa: a cohort study.

Josephine Birungi [1,2] *, Sokoine L Kivuyo[3] *, Anupam Garrib [4] *, Levicatus Mugenyi [1], Gerald Mutungi [5], Ivan Namakoola [2], Janneth Mghamba [6], Kaushik Ramaiya [7], Duolao Wang [4], Sarah Maongezi [6], Joshua Musinguzi [5], Kenneth Mugisha [1], Bernard Micheal Etukoit [1], Ayoub Kakande[2], Louis Niessen [4], Joseph Okebe [4], Tinevimbo Shiri [4], Meshack Shimwela [8], Janet Lutale [9], Geoff Gill [4], Nelson K Sewankambo [10], Peter G Smith [11], Moffat J Nyirenda [2, 12], Sayoki Mfinanga [3,4+], Shabbar Jaffar [4+]

* contributed equally (first authors)
+ contributed equally (senior authors)

1. The AIDS Support Organisation, Mulago Hospital Complex, Kampala, Uganda
2. Medical Research Council/Uganda Virus Research Institute & London School of Hygiene and Tropical Medicine (MRC/UVRI & LSHTM), Uganda Research Unit
3. National Institutes for Medical Research, Dar es Salaam, Tanzania
4. Liverpool School of Tropical Medicine, Pembroke Place, Liverpool, L3 5QA
5. Non-Communicable Diseases and AIDS Control Programmes, Ministry of Health, Uganda
6. Directors Office, Ministry of Health, Community Development, Gender, Elderly and Children, Tanzania.
7. Hindu Mandal Hospital, Dar es Salaam, Tanzania
8. Amana Regional Referral Hospital, Dar es Salaam, Tanzania
9. Muhimbili University of Health and Allied Sciences, Dar es Salaam, Tanzania
10. Makerere University College of Health Sciences, Kampala, Uganda
11. MRC Tropical Epidemiology Group, London School of Hygiene & Tropical Medicine, Keppel Street, London WC1E 7HT
12. Medical Research Council / Uganda Virus Research Institute at the London School of Hygiene & Tropical Medicine Uganda Research Unit, Entebbe, Uganda.

Author for Correspondance: Prof Shabbar Jaffar, shabbar.jaffar@lstmed.ac.uk

For peer review only

Author contributions: SJ, MJN, SMf, AG, GM, JMg, JMu, SMa, JL, GG, NKS and PGS designed the study with support from KR, KM, BE, LN. JB, SLK, IN implemented the study with support from AG, SMf, MJN, JO, TS, MS and KR. SLK and AK managed the data with support from JB and AG. JB, SLK, AG wrote the first drafts and SJ led the later drafts. LM did the statistical analysis with support from DW. JB co-ordinated the study in Uganda and SLK in Tanzania, with support from SJ, AG, MJN and SMf. All authors contributed to various drafts of the paper and saw and approved the final version.

Acknowledgements: We wish to thank Hazel Snell, Erik van Widenfelt, Luis Cuevas, the MOCCA study teams in Tanzania and Uganda and the health facility teams who supported the study and provided patient care, and Drs Mina Nakawuka Ssali and Martha Nabbada from the Ministry of Health in Uganda for their time and support, and Ayubu Masasi, Eric Mgina, Micheal Mubiru and Sebastian Owilla Kidega on data management.

Funding: This research was funded by the National Institute for Health Research (NIHR) (project reference 16/137/87) using UK aid from the UK Government to support global health research. The views expressed in this publication are those of the author(s) and not necessarily those of the NIHR or the UK Department of Health and Social Care.

Conflicts of interest: The authors do not declare any conflict of interest

Data availability statement: No additional data are available.

For peer review only

ABSTRACT

Background. HIV, diabetes and hypertension have a high disease burden in sub-Saharan Africa. Health care is organised in separate clinics, which may be inefficient. In a cohort study, we evaluated integrated management of these conditions from a single chronic care clinic.

Objectives: To determined the feasibility and acceptability of integrated management of chronic conditions in terms of retention in care and clinical indicators.

Design and Setting. Prospective cohort study comprising patients attending 10 health facilities offering primary care in Dar es Salaam and Kampala.

Intervention: Clinics within health facilities were set up to provide integrated care. Patients with either HIV, diabetes or hypertension had the same waiting areas, the same pharmacy, were seen by the same clinical staff, had similar provision of adherence counselling and tracking if they failed to attend appointments.

Primary outcome measures: Retention in care, plasma viral load.

Findings.
Between 5th August 2018 and 21st May 2019, 2,640 patients were screened of whom 2,273 (86%) were enrolled into integrated care (832 with HIV-infection, 313 with diabetes, 546 with hypertension and 582 with multiple conditions). They were followed up to 30th January 2020. Overall, 1615/2273 (71.1%) were female and 1689/2266 (74.5%) had been in care for 6 months or more. The proportions of people retained in care were 686/832 (82.5%, 95% CI 79.9%, 85.1%) among those with HIV-infection, 266/313 (85.0%, 95% CI 81.1%, 89.0%) among those with diabetes, 430/546 (78.8%, 95% CI 75.4%, 82.3%) among those with hypertension and 529/582 (90.9%, 95% CI 88.6, 93.3) among those with multimorbidity. Among those with HIV-infection, the proportion with plasma viral load <100 copies per ml was 423/478 (88.5%).

Conclusion
Integrated management of chronic diseases is a feasible strategy for the control of HIV, diabetes and hypertension in Africa and needs evaluation in a comparative study.

Strengths and limitations

- Study is multi-site and is largest ever conducted on integrating chronic care for HIV, diabetes and management in Africa.

- Study was done in close to real-life health service conditions and so findings likely to be generalisable.

- The study was intended to determine feasibility and early clinical outcomes and does not have a comparison group.

For peer review only

BACKGROUND

The prevalence of diabetes and hypertension has risen sharply in Africa, where these conditions are now responsible for around two million premature adult deaths annually [1-6]. There are effective treatments for diabetes and hypertension, but only 10-20% of persons living with these conditions are thought to be in regular care[1, 7, 8], largely because service provision is limited [9-11]. Identifying approaches to increase access to health services for the management of diabetes and hypertension for populations in Africa represents one of the greatest public health challenges of our time.

Health services in most African countries are generally designed for dealing with acute infections and experience of chronic care management is limited [12], except for HIV-infection which, like other chronic diseases, requires life-long management. In Eastern and Southern Africa 83% of people who know they have HIV-infection are in regular care, with 90% of these virally suppressed[13], compared to a retention rate of about 50% a decade or so ago[14, 15]. HIV associated mortality has declined from over 2 million a year to less than half a million a year [13] and hypertension and diabetes will need to achieve similar levels of retention and disease control to achieve the large declines in mortality needed to meet the Sustainable Development Goals[16].

One reason why HIV services have been successful might be because they have been organised in stand-alone clinics in most African countries from shortly after the time that effective anti-retroviral drugs became available. People living with HIV-infection are managed in separate primary care clinics, with separate drug and diagnostic procurement channels and a separate funding stream. Now that the burden of other chronic conditions is a rapidly increasing public health problem, how should African health systems organise their services?

Managing multiple chronic conditions in the same clinics could reduce the duplication of health care resources and, crucially, could enable the learning acquired by HIV treatment programmes to be applied to the control of other chronic conditions. However, there is little evidence to support this in public health facilities [17, 18]. Integrated management could deter people with diabetes and hypertension in seeking care because of the stigma associated with HIV-infection. There is also a danger, that expanding the focus of health care provision in relatively weak health systems, could put at risk the gains achieved by HIV control programmes. In Tanzania and Uganda, working in close collaboration with policy makers and senior disease control managers, we conducted a cohort study of the integrated care for HIV-infection, diabetes and hypertension and evaluated its feasibility and acceptability in terms of retention in care and clinical indicators including control of blood pressure and fasting plasma glucose.

Methods

For peer review only - http://bmjopen.bmj.com/site/about/guidelines.xhtml

Study design and setting.

We conducted a prospective cohort study, known as the MOCCA study (Management of Chronic Conditions in Africa). We provided integrated care at 10 health facilities that were offering primary health care services, 5 in the Dar es Salaam region in Tanzania and 5 in and around Kampala, Uganda and followed up a cohort of participants over time to determine the feasibility and acceptability of this approach in terms of retention in care and clinical indicators. Appendix 1 describes the health facilities included.

The facilities were serving largely urban and peri-urban populations and chosen purposely. Eight were government run and 2 were run by non-government organisations. They ranged from regional hospitals through to smaller health centres and dispensaries [19, 20]. The larger facilities operated stand-alone separate clinics for diabetes and hypertension, while in the small facilities, patients with diabetes or hypertension were usually seen in the outpatient clinic. Each facility had a HIV clinic run separately from the rest of the clinical services. Patients attending HIV services had their own reception and waiting area, were seen by dedicated clinical staff, and had a separate pharmacy. Medical records, track and trace procedures, and appointments also all differed from systems employed in other clinics in the facility.

What was integrated care? Integration involved the formation of a single clinic for the management of patients with HIV-infection, diabetes or hypertension or combinations of these conditions. All patients were seen by the same clinical staff. They shared a single reception, waiting areas and pharmacy. For patients with diabetes or hypertension, systems for tracking and follow up of patients, recording of clinical notes, counselling, appointments and registrations were mostly set up from new and aligned with those available for people living with HIV, such that all patients were managed in a similar way regardless of their condition.

How was integrated care implemented?
In Uganda the first person received integrated care on the 5th October 2018 and recruitment finished on the 5th of April 2019. The final follow-up was on the 20th December 2019. In Tanzania, the first person received integrated care on 20th February 2019. Recruitment finished on 23rd June 2019. The final follow-up was on 30 January 2020.

We conducted assessments of each facility using a modified WHO Service Availability and Readiness Assessment (SARA) tool and reviewed routine data from the Health Management Information Systems (District Health Information System 2). This provided us with knowledge on the clinical infrastructure, availability of medicines, frequency and nature of patient attendance for assessments.

The integrated care clinic was set up to run separately as a stand-alone clinic at each facility so that patients who did not want integrated care would be able to continue with usual clinics. Organisation of the integrated care clinic, including the frequency of clinic days, was done by health facility managers. Where possible the integrated clinic was run on a different day from the HIV and hypertension or diabetes standalone clinics. In those facilities where

For peer review only

this was not possible, it was run in a separate room away from the main standalone clinics. The integrated clinic was held initially on one day a week at all facilities and later increased to 2 days a week as the numbers of recruited participants increased. Clinical management was done by health care staff employed by the facilities and in accordance with local clinical practice and national guidelines.

The health care staff received refresher training in all three chronic health conditions to ensure a common level of understanding of clinical management. Certified national trainers conducted training in both countries. The training comprised a combination of two days of classroom sessions and on-the-job training. The doctors specialised in HIV and in non-communicable disease management conducted joint clinics for up to one month until they felt comfortable to manage all three conditions. Classroom training included role play of different scenarios.

Counselling was generally not available for diabetes and hypertension management in either country prior to the study but was available routinely for patients with HIV-infection. We therefore provided training to nurses and counsellors on diabetes and hypertension, focussed on the need for high treatment adherence to prevent complications. It comprised one day of classroom training followed by on-the-job training for up to 4 weeks, as done for clinicians, and mentorship.

Prior to the study, HIV clinics had a system of tracking patients who did not attend a scheduled appointment. This generally involved contacting patients by phone if they missed an appointment, to determine the reason for the missed appointment and to reschedule. Such practices were not in place for people with either diabetes or hypertension. Therefore, we extended the system of tracking patients to cover all 3 conditions.

All health facility staff received training on the protocol and research methods. This was approximately one-day of classroom training, followed by on-the-job training.

Data collection: At each clinic, the research team employed one data clerk and one research nurse to inform patients about the research, collect research data and take informed consent. In addition, small amounts of salary (salary top-ups) were provided to selected facility staff working with study participants and to the head nurse responsible for the facility operations including overseeing the logistical set up of integrated care activities. Research staff worked alongside the health facility staff.

Selection of participants. In each disease-specific clinic a health talk was given to introduce the study. The number of patients with diabetes or with multiple conditions was very few. Therefore, all persons presenting with diabetes or with two or all 3 of our target conditions were invited to join the study. Both existing and newly diagnosed patients were enrolled. In the larger facilities (n=3, all in Tanzania), the number of people in care with HIV-infection or with hypertension was large and so we sampled systematically (usually every 20th patient) until the clinic ended for the day. In the remaining clinics, where patient load was lower and it was difficult to ascertain the order that patients had come to the facility, we enrolled patients who approached study staff to find out more, or clinicians approached patients and

For peer review only

invited them to be screened for the study. On most clinic days, patients who had recently arrived at the facility came forward for enrolment, and those who had nearly completed their consultations for the day and were close to leaving the facility for home, were reluctant to come forward.

Provision of medicines for hypertension and diabetes

Before the study started, we recognised that supply of medicines for diabetes and hypertension was erratic in both countries. In Uganda, medicines for these conditions were normally provided free but shortages were common. In Tanzania, most patients were required to purchase medicines, and some had health insurance. We have discussed elsewhere the ethical challenges of conducting research of this kind in situations of limited medicines supply[21].

To ensure a reliable supply of drugs, we purchased back-up supplies of basic drugs for diabetes and hypertension in both Tanzania and Uganda and supplemented patient supplies when the health service stock ran out. The medicines supplied were predominantly first line drugs and differed between countries depending on what was needed.

Due to the limited supply of medicines for diabetes and hypertension in Uganda, patients at one of the study facilities had organised themselves into a group that met and pooled funds on a monthly basis. These funds were used to purchase the medicines when the health facility ran out of stock. At this facility, we offered minimal buffer supplies compared to other facilities.

Measurements.

Retention was assessed through routine attendance at clinics. Patients who failed to attend an appointment were phoned to establish reasons for not attending and to reschedule. Three attempts were made to contact the patient by phone in the month following non-attendance at an appointment. A patient was regarded as lost to follow up if they had not attended an appointment within the last 6 months.

Blood pressure was measured using the Omron M6 comfort (OMRON Healthcare, Japan) in Uganda and in 3 of the facilities in Tanzania; Suresign SLD3-107 (CIGA Healthcare Ltd, UK) was used in the remaining facilities. We measured blood pressure at 3 time points in the sitting position, 5 minutes apart, and took the average of the three readings as the pressure. Hypertension was defined as a systolic pressure ≥140mmHg and/or a diastolic pressure ≥90mmHg and/or use of antihypertensive medication.

Plasma viral load testing was done by the Central Public Health laboratories in Tanzania and Uganda using the Cobas 8800 system (Roche). In Uganda, if the viral load result from the central laboratory was not available or was more than 6 months old, a test was done at the MRC/UVRI/LSHTM Uganda laboratory using Cobas Ampliprep. In both countries HbA1c testing was not routinely available in the public health system, and this was done by the study. In Uganda, HbA1c was tested by Cobas 6000 (Roche, Switzerland) at the MRC/UVRI/LSHTM Uganda laboratory, and in Tanzania at the Shree Hindu Mandal Hospital laboratory using a Siemens DCA Vantage (Siemens Healthcare Diagnostics, Germany).

For peer review only - http://bmjopen.bmj.com/site/about/guidelines.xhtml

Fasting blood glucose was done using a point of care test in Tanzania (GlucoDr. Auto AGM-4000 and (all Medicus Co, Republic of Korea) and in Uganda Contour Plus (Bayer, Germany).

In Tanzania weight and height were measured using SECA RGZ-160 (SECA, GmbH & Co, Germany). In Uganda we used an ADE spring scale (kg, 1-150) for weight measurement and SECA 213 portable stadiometer (SECA, German) for height measurement. Weight and height were measured to the nearest ±0.1 kg and ±0.1 cm, respectively, and body mass index (BMI) was calculated as weight/height^2 (kg/m^2).

Patient and Public involvement

The concept of integrated care was developed in partnership with senior policy makers. In each country, we held planning meetings with the Ministry of Health and non-governmental partners at national and local level, with senior clinicians and hospital managers and with patient leaders and community representatives. National steering committees were formed, comprising representatives from these stakeholders together with the researchers and these committees contributed to the research strategy and guided its implementation. We also formed an international steering committee, that included 4 senior researchers and 4 independent researchers, which had oversight of the whole programme. We also held investigator meetings involving all of the partners and these included patient representatives and policy-makers.

Statistical analysis

The measure of retention used was defined as number in care at study end, calculated as the number enrolled into integrated care minus any deaths, losses-to-follow-up, transfers away from the health facility and withdrawals of consent.

Categorical variables were summarised using frequencies and proportions and compared using Chi-square tests. Continuous variables were summarised using means or medians and compared by a t-test or a Wilcoxon test, as appropriate. Generalized Estimating Equations (GEE) were used to account for clustering at the health facility level. Kaplan Meier curves and Cox regression with frailties (to adjust for clustering) were used to analyse retention times. The time was calculated as number of months from first attendance at the integrated clinic to the earliest of the end of the study, loss to follow up, death, withdrawal, or transfer-out. The analysis was done using STATA version 15.

Ethics clearance

The study received ethical clearance from TASO Research Ethics Committee and Uganda National Council for Science and Technology (UNCST) in Uganda (Reference TASO REC/015/18-UG-REC-009), National Health Research Ethics Review Sub-Committee in Tanzania (Reference NIMR/HQ/R.8a/Vol. IX/2793), and the Liverpool School of Tropical Medicine in the UK (Reference 18-044).

RESULTS

For peer review only - http://bmjopen.bmj.com/site/about/guidelines.xhtml

Figure 1 shows the consort chart. Of 2640 patients assessed for eligibility, 102 (3.9%) did not meet eligibility criteria (34 were sick and requiring urgent medical attention or hospital admission, 50 were planning to move out of the area, 13 were pregnant and receiving care from antenatal care services, 5 other reasons), 19 (0.7%) declined and gave no reason, 51(1.9%) wanted more time to decide (mostly to discuss with their family), 34 (1.3%) were approached just as they had finished consultation and were leaving the clinic and 18 (0.7%) gave other reasons (mostly that they needed to be in specialist care).

Thus, of all those initially assessed for eligibility 91.5% (2416/2640) were invited to join integrated care. This invitation was given at their current clinics and they were asked to return at their next visit to the integrated care clinic. Six percent (143/2416) did not return and thus 2,273/2640 (86.1%) were enrolled into integrated care, 927 in Tanzania and 1346 in Uganda.

Table 1 shows the characteristics of the participants at the start of integrated care, for each clinical condition. Overall, 74.5% (1689/2266) reported that they had been in care for 6-months or more prior to the start of the study. For people with multiple conditions, previous time in care was substantially greater than for those with single conditions. Overall, 71.1% (1615/2273) of those included in the study were female.

Median age varied substantially between the different conditions, with that for those living with HIV-infection being more than 10 years younger than participants in the other groups (p=0.0001, Kruskal Wallis test). Obesity was more common among participants with diabetes, hypertension or multimorbidity than among participants with HIV (504/1441 (35.0%) versus 116/832 (13.9%), risk ratio adjusted for clustering: 2.50, 95% CI: 1.88, 3.31, p<0.001). It was also substantially higher in women than in men (554/1615 (34.3%) vs 66/658 (10.0%), adjusted risk ratio: 3.40, 95% CI: 2.63– 4.41, p<0.001).

Table 2 shows the biomedical indicators as measured at baseline on the day of integration or within the previous 6 months. The majority did not reach target blood pressure and glycaemia control levels, whether they had a single condition or multimorbidity. When participants who reported that they had been in care for less than 6 months (i.e. patients who had newly started therapy) were excluded, 509/882 (57.7%) of those with hypertension had a blood pressure ≥140/90 mmHg, 355/508 (69.9%) of those with diabetes had a fasting blood glucose ≥7.0 mmol/L and 72/438 (16.4%) of those with HIV-infection had a plasma viral load ≥100 copies per ml.

The proportions alive and retained in care were high among all participants (Table 3). The overall proportion alive and in care among participants with a single condition was 1382/1691 (81.7%, 95% CI: 79.9% - 83.6%), which did not differ significantly from the proportion with multimorbidity (adjusted risk ratio 0.91, 95% CI: 0.82, 1.00, p=0.061).

Figure 2 shows the survival probability curves of being alive and in care over time. After adjusting for clustering, the probability (95% CI) of being retained and alive in care at 12 months was 0.74 (0.66 – 0.80) among participants with HIV alone, 0.74 (0.66– 0.81) among those with diabetes alone, 0.70 (0.64 – 0.76) among those with hypertension alone and 0.84 (0.79 – 0.88) among those with multimorbidity (p=0.008).

For peer review only - http://bmjopen.bmj.com/site/about/guidelines.xhtml

Table 4 shows the biomedical indicators of participants at the final follow-up. The proportions of patients living with HIV-infection with viral suppression were high, but the proportions of those with hypertension achieving blood pressure less than 140/90 mmHg and those with diabetes achieving glycaemia <7 mmol/L were substantially lower. However, blood pressure, fasting glucose and plasma viral load were all better controlled at study end than at baseline (Table 5), even when participants who had been in care for less than 6 months, and would have been newly started on therapy were excluded. After adjusting for clustering, among patients with HIV, the proportion (95% CI) with plasma viral load of <100 copies per ml was 88.5% (85.7%-91.4%) and those with plasma viral load <1000 copies per ml was 96.0% (94.3%-97.8%).

DISCUSSION

This study shows that integrating care for HIV-infection, diabetes and hypertension achieved high levels of retention in care for people living with diabetes or hypertension in Africa. Crucially, this improvement did not appear to be at the expense of HIV control, as retention in all 3 groups of people with single conditions was similar, and the proportion with an HIV viral suppression of less than a 100 copies per ml was close to 90%.

Thus, our study suggests that an integrated approach could achieve excellent retention for diabetes and hypertension in an integrated clinic, as has been achieved for people living with HIV-infection where retention exceeds 80% today, up from about 50% a decade or so ago[13-15].

The retention rate for people with diabetes or hypertension observed in our study was higher than that published from some other studies, which involved studies of single diseases, including those where diagnostic and treatment services were enhanced and, for example, treatments were provided for free[22-24]. In our study, we put in place basic measures to support participants with diabetes and hypertension including minimal access to basic medicines, counselling and telephone reminders for those who missed appointments. However, these measures were no more than those available to people in care with HIV-infection and no more than those available to people in research studies of hypertension and diabetes. No financial or other incentives were provided in our study and participants were managed by routine health care staff. Analysis of the costs of this package will be done separately but generic medicines for diabetes and hypertension and associated monitoring costs are substantially less than those for HIV-infection and so we anticipate that the model will be highly cost-effective.

In a large study in Malawi conducted by members of our team almost a decade ago, people identified with diabetes and hypertension in the community were referred into care with a dedicated medical officer and access to a free and reliable supply of medicines and diagnostics. However, at 12 months retention in care was 27% , suggesting that control of diabetes and hypertension when these conditions are identified through routine screening (i.e. are asymptomatic) might be a challenge[23, 25]. A study from Kwazulu-Natal in South Africa reported similar findings [26]. However, other studies that provided a good level of

For peer review only

care and that have involved patients who know they have diabetes and hypertension, have also struggled to achieve good levels of retention[22].

Why did our study achieve high retention? For people with multiple conditions, a huge advantage of integrated care is that they only need to attend one clinic appointment, but in our study retention was also high for people with single conditions. It is possible that when services were integrated and the lessons learned in managing HIV-infection with respect to counselling, treatment adherence and the provision of a more streamlined service, were applied by health care staff to diabetes and hypertension management, patients responded to the changes in service delivery. The mixing of patients with HIV-infection with those living with diabetes and hypertension may also have led to the sharing of experiences of living with a long-term condition, which could have brought further benefit.

We knew that integration of services would involve expansion of care responsibilities of health facility staff, and feared that this would adversely impact HIV care outcomes [17, 27, 28]. However, this did not happen. HIV viral suppression rates were high at enrolment and were slightly higher at the end of the study. There is an increasing body of literature on integrating screening, diagnosis and treatment for non-communicable diseases into established HIV services.[17, 29, 30] As far as we are aware, ours is the first study to test integration of services fully so that any person with one or more of the 3 target conditions could be managed in one clinic[29, 31].

We had feared that stigma may put off patients with diabetes and hypertension from attending a joint service with patients living with HIV. However, none of the patients who declined to join cited stigma. It is possible that the 41 patients who declined without giving a reason or who deferred their decision, did so because of the stigma. Nonetheless, these numbers of refusals are relatively small, and very few study participants withdrew over time. HIV has a special status in almost all African countries, which may have contributed to the stigma associated with this disease. Our study suggests that HIV-infection can be managed in the same space as other conditions – i.e. that HIV can be considered like any other disease or condition – and this will not make a difference to the outcome for the patient living with HIV-infection.

Although we achieved high rates of retention, and blood pressure and glycaemia control improved over time, a high percentage of those with diabetes or hypertension continued to have uncontrolled blood pressure and uncontrolled glycaemia at the end of the study, in contrast to the situation with HIV for which the vast majority maintained excellent control of viraemia. In the case of diabetes, this is complicated by a lack of access to adequate monitoring. Access to HbA1c testing was a challenge we faced in this study, and is a challenge across Africa[32]. The next step in this story is to identify and test interventions that improve blood pressure and diabetes control (among people in care) and take this control to levels achieved by HIV programmes in order to drive down mortality from non-communicable diseases.

Large numbers of people in high income countries are now living with multiple conditions – so called multimorbidity - and this number is expected to continue to rise sharply in Africa, if it has not done so. In our study, individuals with more than one condition, who would

For peer review only - http://bmjopen.bmj.com/site/about/guidelines.xhtml

normally attend multiple different clinics, appeared to have a higher retention in care than people with a single condition but their clinical indicators at study end were no different from those with a single condition. While it is not surprising that their retention should be high, the lack of superiority in their clinical indicators from those with a single condition, suggest that greater efforts will be needed to improve clinical outcomes.

Studies that involve re-organisation of care provision represent huge change and challenges for health services, communities and patients. They are not possible without partnerships based on an ethos of equality and openness and without the building of trust between researchers and these stakeholders. It is essential that research of this nature allows time and resource for the working together of different groups of researchers, health care providers and public health authorities, patients and communities.

Strengths and Limitations Although the evidence generated by our study is much greater than that available to most policy makers, and separate clinics for each chronic condition are not sustainable for Ministries of Health, changes to health policy that would affect tens of millions of people living with chronic conditions, requires robust evidence. Our study was large, comprising over 2000 participants with different chronic conditions. However, it was a pilot study and did not have a comparison group. It was done in just 10 health facilities, 5 in Tanzania and 5 in Uganda. In some health facilities, the number of people presenting with HIV or hypertension was high and the facilities had no system for recording the order in which patients presented and so we were unable to choose participants systematically but selected them purposively. This could have introduced selection bias.

Evidence is needed from large randomised trials with control groups comprising current standard care as provided in stand-alone clinics [17], with replication in multiple settings and over a longer duration of follow-up and with systematic or random sampling of participants to reduce the possibility bias. We also need more evidence on why integrated care works for different groups of people either with single or with multiple conditions.

For peer review only

Table 1. Baseline characteristics of patients studied.

**Number recruited into integrated care**		**HIV only**	**Diabetes only**	**Hypertension only**	**Multiple conditions**
	**Total**	**832**	**313**	**546**	**582**
	In Tanzania	352	164	160	251
	In Uganda	480	149	386	331
**Type of health facility**					
	Large public facilities	235	81	153	184
	Small public facilities	409	155	282	217
	NGO facilities	188	77	111	181
**Time in care prior to enrolment, number (%) ***					
	< 6 months	310 (37.4)	63 (20.3)	165 (30.3)	39 (6.7)
	6-11 months	41 (5.0)	28 (9.0)	42 (7.7)	22 (3.8)
	12-59 months	167 (20.1)	98 (31.6)	172 (31.6)	135 (23.2)
	>=60 months	311 (37.5)	121 (39.0)	166 (30.5)	386 (66.3)
**Median (range) months in care**		26.1 (0.0 – 343.4)	38.3 (0.0 – 431.6)	26.4 (0.0 – 566.2)	106 (0.0 – 534.8)
**Female, number (%)**		571 (68.6)	208 (66.5)	399 (73.1)	437 (75.1)
**Median age, (range)**		38 (18 – 78)	49 (19 – 77)	56 (27 – 86)	55 (21 – 85)
**BMI, number (%)**					
	<25	531 (63.8)	118 (37.7)	175 (32.1)	163 (28.0)
	25-29.9	185 (22.2)	98 (31.3)	181 (33.2)	202 (34.7)
	≥30	116 (13.9)	97 (31.0)	190 (34.8)	217 (37.3)
**Median BMI (range) in men**		21.1 (14.8 – 35.6)	24.3 (15.1 – 37.0)	25.1 (16.7 – 41.6)	26.0 (17.0 – 53.1)
**Median BMI (range) in women**		24.4 (13.2 – 48.9)	28.5 (15.4 – 48.2)	28.8 (16.0 – 65.2)	28.8 (15.8 – 57.9)

For peer review only

NOTES

* missing for 7 participants (3 with HIV-infection, 3 diabetes and 1 hypertension).

Table 2. Biomedical indicators at baseline.

	Patients who have a single condition occurring alone	The condition occurs with other conditions, causing multimorbidity (only among those with the condition)	Both combined
**AMONGST PATIENTS WITH HYPERTENSION**			
**Blood pressure (mmHg), number (%)**	**N=546**	**N=539**	**N=1085**
**≥140/90**	342 (62.6)	327 (60.7)	669 (61.7)
**<140/90**	204 (37.4)	212 (39.3)	416 (38.3)
**≥180/120**	42 (7.7)	64 (11.9)	106 (9.8)
**<180/120**	504 (92.3)	475 (88.1)	979 (90.2)
**Systolic BP mmHg, mean (range)**	144.4 (83.0 – 257.7)	145.1 (68.7 – 271.0)	144.7 (68.7 – 271.0)
**Diastolic BP mmHg, mean (range)**	90.2 (43.3 – 163.0)	90.3 (54.7 – 167.3)	90.2 (43.3 – 167.3)
**AMONGST PATIENTS WITH DIABETES**			
**Fasting glucose (mmol/L), number (%)**	**N=228**	**N=281**	**N=509**
<6.1	34 (14.9)	62 (22.1)	96 (18.9)
6.1–6.9	17 (7.5)	32 (11.4)	49 (9.6)
≥7.0	177 (77.6)	187 (66.6)	364 (71.5)
**Fasting glucose, mean (range) mmol/L**	11.5 (2.9 – 29.3)	9.8 (2.3 – 28.2)	10.5 (2.3 – 29.3)
**HbA1c, number (%) ***	**N=124**	**N=206**	**N=330**
(<6.0%	4 (3.2)	25 (12.1)	29 (8.8)
6.0–6.4%	7 (5.7)	22 (10.7)	29 (8.8)
≥6.5%	113 (91.1)	159 (77.2)	272 (82.4)
**HbA1c, mean (range) percent**	10.1 (4.8 – 16.9)	8.8 (4.8 – 16.5)	9.3 (4.8 – 16.9)
**AMONGST PATIENTS LIVING WITH HIV**			
**HIV plasma viral load, number (%) ***	**N= 493**	**N= 153**	**N= 646**
<100 copies/ml	306 (62.1)	131 (85.6)	437 (67.7)
100-999 copies/ml	68 (13.8)	14 (9.2)	82 (12.7)
≥1000 copies/ml	119 (24.1)	8 (5.2)	127 (19.7)

* measured at any time within the previous 6 months. Overall for the proportion with plasma viral load <100 copies/ml, the 95% CI was 64.1%, 71.4% and 80.3% (95% CI 77.3%-83.5%) had a plasma viral load < 1000 copies per ml.

Note: Blood pressure was measured at baseline by research staff among 1,187/1188 (99.9%) participants who were not known previously to be hypertensive. Of these, the proportion who were detected with a blood pressure of 140/90 mmHg or higher was 80/831 (9.6%) among those with HIV-infection alone, 47/313 (15.0%) among those with diabetes alone and 6/43 (14.0%) among those with HIV-infection and diabetes. Of note, among all HIV-infected participants, blood pressure was 180/120 mmHg or higher among 45/1126 (4.0%).

For peer review only

For peer review only - http://bmjopen.bmj.com/site/about/guidelines.xhtml

Table 3. Retention in integrated care and clinical outcomes according to condition.

Overall	HIV only	Diabetes only	Hypertension only	Multiple conditions
**Number enrolled into integration**	**832**	**313**	**546**	**582**
Median follow-up (inter-quartile range)	8.2 (6.9 – 10.4)	8.0 (6.9 – 9.4)	8.2 (6.9 – 10.1)	8.2 (7.2 – 9.8)
Retained in care to the end of the study n [% (95%CI)]*	n. 686 82.5 (79.9 –85.1)	n. 266 85.0 (81.1 – 89.0)	n. 430 78.8 (75.4 – 82.3)	n. 529 90.9 (88.6–93.3)
Rate per 100 person years (95% CI)*	75.0 (70.6, 78.7)	77.7 (70.4, 83.3)	69.9 (63.5, 74.6)	87.1 (83.2, 90.2)

* adjusted for clustering at health facility level

Notes: 85 persons were newly diagnosed with multiple conditions over the course of follow-up. Of participants with HIV alone, 5 were diagnosed with diabetes and 33 with hypertension. Of those with diabetes, 3 tested positive for HIV and 23 were diagnosed with hypertension. Of those with hypertension, 1 tested positive for HIV and 9 were diagnosed with diabetes. Eleven persons with dual conditions were diagnosed with a 3rd condition.

For peer review only

Table 4. Biomedical indicators at study end.

	Patients who have a single condition occurring alone	The condition occurs with other conditions, causing multimorbidity	Both combined
**Blood pressure (mmHg), number (%)**	**N=415**	**N=469**	**N=884**
≥140/90	183 (44.1)	225 (48.0)	408 (46.2)
<140/90	232 (55.9)	244 (51.2)	476 (53.9)
≥180/120	7 (1.7)	25 (5.3)	32 (3.6)
<180/120	408 (98.3)	444 (94.7)	852 (96.4)
**Systolic BP mmHg, mean (range)**	133.9 (26.0 – 228)	136.7 (90.0 – 227)	135.4 (26 – 228)
**Diastolic BP mmHg, mean (range)**	83.9 (53 – 130)	85.6 (44 – 185)	84.8 (44 – 185)
**Fasting glucose (mmol/L), number (%)**	**N=189**	**N=242**	**N=431**
<6.1	36 (19.1)	69 (28.5)	105 (24.4)
6.1–6.9	28 (14.8)	33 (13.6)	61 (14.2)
≥7.0	125 (66.1)	140 (57.9)	265 (61.5)
**Fasting glucose, mean (range) mmol/L**	9.7 (3.6 – 28.5)	8.3 (3.6 – 19.8)	8.9 (3.6 – 28.5)
**HbA1c, number (%).**	**N=84**	**N=140**	**N=224**
(<6%	17 (20.2)	31 (22.1)	48 (21.4)
6.0-6.4%	4 (4.8)	15 (10.7)	19 (8.5)
≥6.5%	63 (75.0)	94 (67.1)	157 (70.1)
**HbA1c, mean (range) percent**	7.6 (0.6 – 13.5)	7.3 (0.6 – 15.9)	7.4 (0.6 – 15.9)
**HIV plasma viral load, number (%) ***	**N=322**	**N=156**	**N=478**
<100 copies/ml	278 (86.3)	145 (93.0)	423 (88.5)
100-999 copies/ml	27 (8.4)	9 (5.8)	36 (7.5)
>=1000 copies/ml	17 (5.3)	2 (1.3)	19 (4.0)

* The 95% CI for plasma viral load<100 copies/ml for both groups combined was 85.7, 91.4%). The proportion with plasma viral load<1000 copies per ml was 96.0% (95% CI 94.3%, 97.8%).

Note: 80 persons were newly diagnosed with 2nd or 3rd conditions over the course of the follow-up (see Table 3). Their final follow-up indicators are not included in the analyses above as these patients may have been newly started on treatment and therefore will not have had time to stabilise.

For peer review only

607 **Table 5. Risk differences (95% CI) between baseline and study end among participants**
608 **living with hypertension, diabetes or HIV-infection.**
609

Clinical condition (either alone or alongside other conditions)	All participants	Excluding participants in care for less 6 months at enrolment
Raised blood pressure (≥140/90 mmHg)	14.8%, (10.5%, 19.2%) (p<0.001)	10.5%, (5.7%, 15.3%) p<0.001).
Raised fasting glucose (≥7 mmol/L)	8.4%, (2.4%, 14.3%) p=0.006)	7.4% (1.0%, 13.7%) p=0.024)
Lack of viral suppression (plasma viral load ≥ 100 copies per ml)	20.8%, (16.0%, 25.5%) p<0.001).	7.3% (2.7%, 11.9%) p=0.002)

610 Notes: the risk difference is calculated as risk estimate at baseline minus risk estimate at study end.
611
612
613
614
615
616
617
618

For peer review only

References

1. Atun, R., J.I. Davies, E.A.M. Gale, T. Barnighausen, D. Beran, A.P. Kengne, N.S. Levitt, F.W. Mangugu, M.J. Nyirenda, G.D. Ogle, K. Ramaiya, N.K. Sewankambo, E. Sobngwi, S. Tesfaye, J.S. Yudkin, S. Basu, C. Bommer, E. Heesemann, J. Manne-Goehler, I. Postolovska, V. Sagalova, S. Vollmer, Z.G. Abbas, B. Ammon, M.T. Angamo, A. Annamreddi, A. Awasthi, S. Besancon, S. Bhadriraju, A. Binagwaho, P.I. Burgess, M.J. Burton, J. Chai, F.P. Chilunga, P. Chipendo, A. Conn, D.R. Joel, A.W. Eagan, C. Gishoma, J. Ho, S. Jong, S.S. Kakarmath, Y. Khan, R. Kharel, M.A. Kyle, S.C. Lee, A. Lichtman, C.P. Malm, M.N. Mbaye, M.A. Muhimpundu, B.M. Mwagomba, K.J. Mwangi, M. Nair, S.P. Niyonsenga, B. Njuguna, O.L.O. Okafor, O. Okunade, P.H. Park, S.D. Pastakia, C. Pekny, A. Reja, C.N. Rotimi, S. Rwunganira, D. Sando, G. Sarriera, A. Sharma, A. Sidibe, E.S. Siraj, A.S. Syed, K. Van Acker, and M. Werfalli, *Diabetes in sub-Saharan Africa: from clinical care to health policy.* Lancet Diabetes Endocrinol, 2017. **5**(8): p. 622-667.
2. Bosu, W.K., S.T. Reilly, J.M.K. Aheto, and E. Zucchelli, *Hypertension in older adults in Africa: A systematic review and meta-analysis.* PLoS One, 2019. **14**(4): p. e0214934.
3. Guwatudde, D., J. Nankya-Mutyoba, R. Kalyesubula, C. Laurence, C. Adebamowo, I. Ajayi, F. Bajunirwe, M. Njelekela, F.S. Chiwanga, T. Reid, J. Volmink, H.O. Adami, M.D. Holmes, and S. Dalal, *The burden of hypertension in sub-Saharan Africa: a four-country cross sectional study.* BMC Public Health, 2015. **15**: p. 1211.
4. Mills, K.T., J.D. Bundy, T.N. Kelly, J.E. Reed, P.M. Kearney, K. Reynolds, J. Chen, and J. He, *Global Disparities of Hypertension Prevalence and Control: A Systematic Analysis of Population-Based Studies From 90 Countries.* Circulation, 2016. **134**(6): p. 441-50.
5. Organisation, W.H., *Descrption status report on non-communicable diseases 2010.* 2011, 2010(Geneva, Switzerland).
6. Sarki, A.M., C.U. Nduka, S. Stranges, N.B. Kandala, and O.A. Uthman, *Prevalence of Hypertension in Low- and Middle-Income Countries: A Systematic Review and Meta-Analysis.* Medicine (Baltimore), 2015. **94**(50): p. e1959.
7. Addo, J., L. Smeeth, and D.A. Leon, *Hypertension in sub-saharan Africa: a systematic review.* Hypertension, 2007. **50**(6): p. 1012-8.
8. Geldsetzer, P., J. Manne-Goehler, M.E. Marcus, C. Ebert, Z. Zhumadilov, C.S. Wesseh, L. Tsabedze, A. Supiyev, L. Sturua, S.K. Bahendeka, A.M. Sibai, S. Quesnel-Crooks, B. Norov, K.J. Mwangi, O. Mwalim, R. Wong-McClure, M.T. Mayige, J.S. Martins, N. Lunet, D. Labadarios, K.B. Karki, G.B. Kagaruki, J.M.A. Jorgensen, N.C. Hwalla, D. Houinato, C. Houehanou, M. Msaidie, D. Guwatudde, M.S. Gurung, G. Gathecha, M. Dorobantu, A. Damasceno, P. Bovet, B.W. Bicaba, K.K. Aryal, G. Andall-Brereton, K. Agoudavi, A. Stokes, J.I. Davies, T. Barnighausen, R. Atun, S. Vollmer, and L.M. Jaacks, *The state of hypertension care in 44 low-income and middle-income countries: a cross-sectional study of nationally representative individual-level data from 1.1 million adults.* Lancet, 2019. **394**(10199): p. 652-662.
9. Katende, D., G. Mutungi, K. Baisley, S. Biraro, E. Ikoona, R. Peck, L. Smeeth, R. Hayes, P. Munderi, and H. Grosskurth, *Readiness of Ugandan health services for the management of outpatients with chronic diseases.* Trop Med Int Health, 2015. **20**(10): p. 1385-95.
10. Peck, R., J. Mghamba, F. Vanobberghen, B. Kavishe, V. Rugarabamu, L. Smeeth, R. Hayes, H. Grosskurth, and S. Kapiga, *Preparedness of Tanzanian health facilities for*

For peer review only - http://bmjopen.bmj.com/site/about/guidelines.xhtml

outpatient primary care of hypertension and diabetes: a cross-sectional survey. Lancet Glob Health, 2014. **2**(5): p. e285-92.

11. Manne-Goehler, J., R. Atun, A. Stokes, A. Goehler, D. Houinato, C. Houehanou, M.M. Hambou, B.L. Mbenza, E. Sobngwi, N. Balde, J.K. Mwangi, G. Gathecha, P.W. Ngugi, C.S. Wesseh, A. Damasceno, N. Lunet, P. Bovet, D. Labadarios, K. Zuma, M. Mayige, G. Kagaruki, K. Ramaiya, K. Agoudavi, D. Guwatudde, S.K. Bahendeka, G. Mutungi, P. Geldsetzer, N.S. Levitt, J.A. Salomon, J.S. Yudkin, S. Vollmer, and T. Barnighausen, *Diabetes diagnosis and care in sub-Saharan Africa: pooled analysis of individual data from 12 countries.* Lancet Diabetes Endocrinol, 2016. **4**(11): p. 903-912.
12. Garrib, A., J. Birungi, S. Lesikari, I. Namakoola, T. Njim, L. Cuevas, L. Niessen, K. Mugisha, G. Mutungi, J. Mghamba, K. Ramaiya, S. Jaffar, S. Mfinanga, and M. Nyirenda, *Integrated care for human immunodeficiency virus, diabetes and hypertension in Africa.* Trans R Soc Trop Med Hyg, 2019. **113**(12): p. 809-812.
13. UNAIDS, *UNAIDS Data 2020*. 2020, UNAIDS: Geneva, Switzerland.
14. Rosen, S., M.P. Fox, and C.J. Gill, *Patient retention in antiretroviral therapy programs in sub-Saharan Africa: a systematic review.* PLoS Med, 2007. **4**(10): p. e298.
15. Rosen, S. and M.P. Fox, *Retention in HIV care between testing and treatment in sub-Saharan Africa: a systematic review.* PLoS Med, 2011. **8**(7): p. e1001056.
16. collaborators, N.C.D.C., *NCD Countdown 2030: pathways to achieving Sustainable Development Goal target 3.4.* Lancet, 2020. **396**(10255): p. 918-934.
17. Haldane, V., H. Legido-Quigley, F.L.H. Chuah, L. Sigfrid, G. Murphy, S.E. Ong, F. Cervero-Liceras, N. Watt, D. Balabanova, S. Hogarth, W. Maimaris, K. Buse, M. McKee, P. Piot, and P. Perel, *Integrating cardiovascular diseases, hypertension, and diabetes with HIV services: a systematic review.* AIDS Care, 2018. **30**(1): p. 103-115.
18. Khabala, K.B., J.K. Edwards, B. Baruani, M. Sirengo, P. Musembi, R.J. Kosgei, K. Walter, J.M. Kibachio, M. Tondoi, H. Ritter, E. Wilkinson, and T. Reid, *Medication Adherence Clubs: a potential solution to managing large numbers of stable patients with multiple chronic diseases in informal settlements.* Trop Med Int Health, 2015. **20**(10): p. 1265-70.
19. Briggs, C.J., P. Capdegelle, and P. Garner, *Strategies for integrating primary health services in middle- and low-income countries: effects on performance, costs and patient outcomes.* Cochrane Database Syst Rev, 2001(4): p. CD003318.
20. Briggs, C.J. and P. Garner, *Strategies for integrating primary health services in middle- and low-income countries at the point of delivery.* Cochrane Database Syst Rev, 2006(2): p. CD003318.
21. Shayo, E., M.C. Van Hout, J. Birungi, A. Garrib, S. Kivuyo, S. Mfinanga, M.J. Nyrienda, I. Namakoola, J. Okebe, K. Ramaiya, M.O. Bachmann, W. Cullen, J.V. Lazarus, G. Gill, T. Shiri, D. Bukenya, H. Snell, M. Nanfuka, L.E. Cuevas, M. Shimwela, G. Mutungi, J. Musinguzi, J. Mghamba, K. Mugisha, S. Jaffar, P.G. Smith, and N.K. Sewankambo, *Ethical issues in intervention studies on the prevention and management of diabetes and hypertension in sub-Saharan Africa.* BMJ Glob Health, 2020. **5**(7).
22. Labhardt, N.D., J.R. Balo, M. Ndam, E. Manga, and B. Stoll, *Improved retention rates with low-cost interventions in hypertension and diabetes management in a rural African environment of nurse-led care: a cluster-randomised trial.* Trop Med Int Health, 2011. **16**(10): p. 1276-84.
23. Musicha, C., A.C. Crampin, N. Kayuni, O. Koole, A. Amberbir, B. Mwagomba, S. Jaffar, and M.J. Nyirenda, *Accessing clinical services and retention in care following*

For peer review only

screening for hypertension and diabetes among Malawian adults: an urban/rural comparison. J Hypertens, 2016. **34**(11): p. 2172-9.
24. Gill, G.V., C. Price, D. Shandu, M. Dedicoat, and D. Wilkinson, *An effective system of nurse-led diabetes care in rural Africa.* Diabet Med, 2008. **25**(5): p. 606-11.
25. Price, A.J., A.C. Crampin, A. Amberbir, N. Kayuni-Chihana, C. Musicha, T. Tafatatha, K. Branson, D.A. Lawlor, E. Mwaiyeghele, L. Nkhwazi, L. Smeeth, N. Pearce, E. Munthali, B.M. Mwagomba, C. Mwansambo, J.R. Glynn, S. Jaffar, and M. Nyirenda, *Prevalence of obesity, hypertension, and diabetes, and cascade of care in sub-Saharan Africa: a cross-sectional, population-based study in rural and urban Malawi.* Lancet Diabetes Endocrinol, 2018. **6**(3): p. 208-222.
26. Siedner, M.J., K. Baisley, J. Orne-Gliemann, D. Pillay, O. Koole, E.B. Wong, P. Matthews, F. Tanser, K. Herbst, T. Barnighausen, and M. Bachmann, *Linkage to primary care after home-based blood pressure screening in rural KwaZulu-Natal, South Africa: a population-based cohort study.* BMJ Open, 2018. **8**(12): p. e023369.
27. Rawat, A., K. Uebel, D. Moore, and A. Yassi, *Integrated HIV-Care Into Primary Health Care Clinics and the Influence on Diabetes and Hypertension Care: An Interrupted Time Series Analysis in Free State, South Africa Over 4 Years.* 2018. **77**(5): p. 476-483.
28. Matanje Mwagomba, B.L., S. Ameh, P. Bongomin, P.A. Juma, R.K. MacKenzie, C. Kyobutungi, N. Lukhele, K.J.M. Mwangi, A. Amberbir, K. Klipstein-Grobusch, F.X. Gomez-Olive, and J. Berman, *Opportunities and challenges for evidence-informed HIV-noncommunicable disease integrated care policies and programs: lessons from Malawi, South Africa, Swaziland and Kenya.* AIDS, 2018. **32 Suppl 1**: p. S21-S32.
29. Duffy, M., B. Ojikutu, S. Andrian, E. Sohng, T. Minior, and L.R. Hirschhorn, *Non-communicable diseases and HIV care and treatment: models of integrated service delivery.* Trop Med Int Health, 2017. **22**(8): p. 926-937.
30. Njuguna, B., S. Vorkoper, P. Patel, M.J.A. Reid, R. Vedanthan, C. Pfaff, P.H. Park, L. Fischer, J. Laktabai, and S.D. Pastakia, *Models of integration of HIV and noncommunicable disease care in sub-Saharan Africa: lessons learned and evidence gaps.* AIDS, 2018. **32 Suppl 1**: p. S33-S42.
31. Wroe, E.B., N. Kalanga, B. Mailosi, S. Mwalwanda, C. Kachimanga, K. Nyangulu, E. Dunbar, L. Kerr, L. Nazimera, and L. Dullie, *Leveraging HIV platforms to work toward comprehensive primary care in rural Malawi: the Integrated Chronic Care Clinic.* Healthc (Amst), 2015. **3**(4): p. 270-6.
32. Park, P.H. and S.D. Pastakia, *Access to Hemoglobin A1c in Rural Africa: A Difficult Reality with Severe Consequences.* J Diabetes Res, 2018. **2018**: p. 6093595.

For peer review only

Legends to Figures

Figure 1. Study schema

Figure 2. Kaplan-Meier curves showing probability proportion of patients of remaining in care according to condition.

For peer review only

For peer review only - http://bmjopen.bmj.com/site/about/guidelines.xhtml

Figure 1:

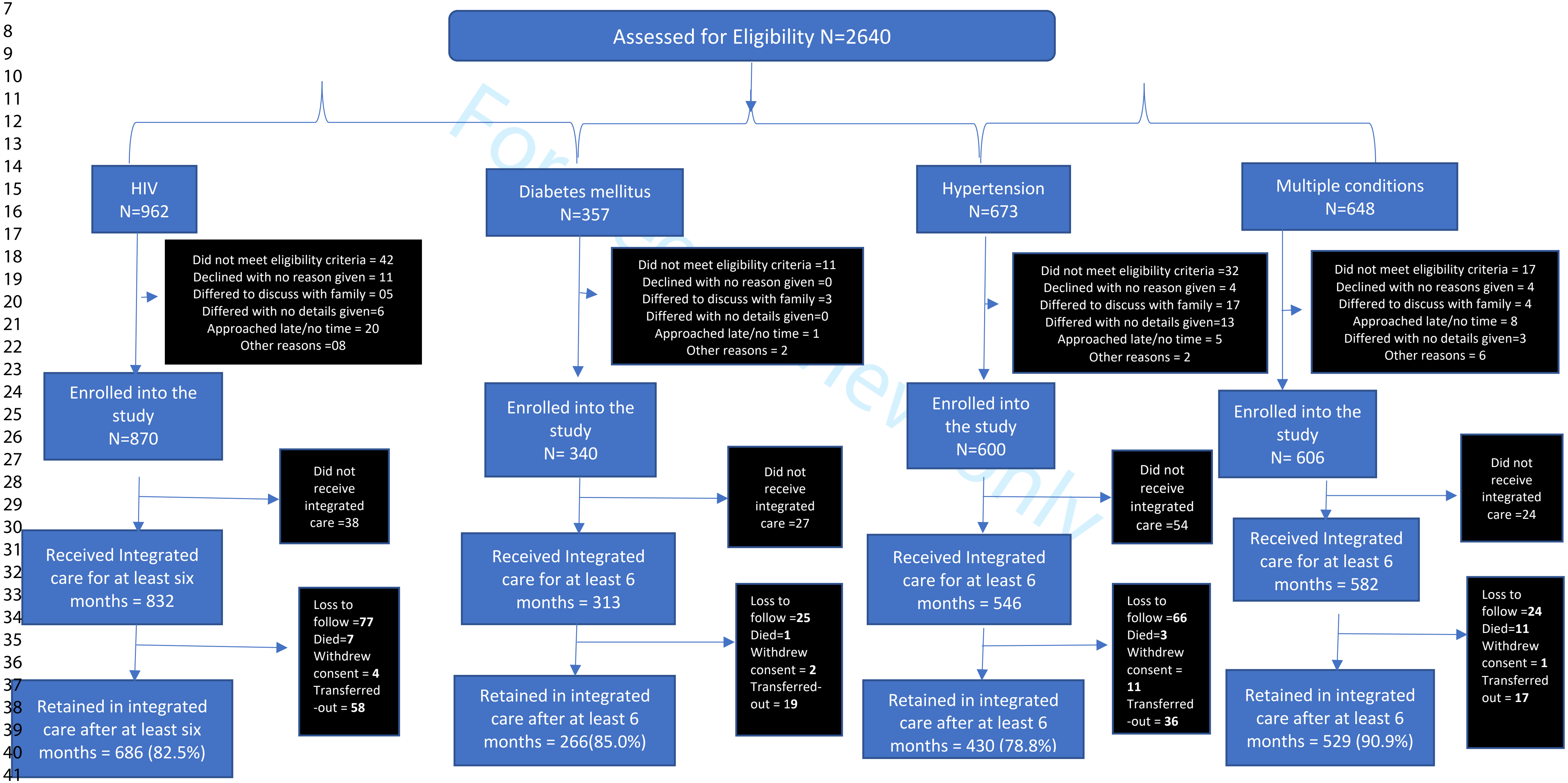
Assessed for Eligibility N=2640
HIV
N=962
Did not meet eligibility criteria = 42
Declined with no reason given = 11
Differed to discuss with family = 05
Differed with no details given=6
Approached late/no time = 20
Other reasons =08
Enrolled into the study
N=870
Did not receive integrated care =38
Received Integrated care for at least six months = 832
Loss to follow =77
Died=7
Withdrew consent = 4
Transferred -out = 58
Retained in integrated care after at least six months = 686 (82.5%)
Diabetes mellitus
N=357
Did not meet eligibility criteria =11
Declined with no reason given =0
Differed to discuss with family =3
Differed with no details given=0
Approached late/no time = 1
Other reasons = 2
Enrolled into the study
N= 340
Did not receive integrated care =27
Received Integrated care for at least 6 months = 313
Loss to follow =25
Died=1
Withdrew consent = 2
Transferred-out = 19
Retained in integrated care after at least 6 months = 266(85.0%)
Hypertension
N=673
Did not meet eligibility criteria =32
Declined with no reason given = 4
Differed to discuss with family = 17
Differed with no details given=13
Approached late/no time = 5
Other reasons = 2
Enrolled into the study
N=600
Did not receive integrated care =54
Received Integrated care for at least 6 months = 546
Loss to follow =66
Died=3
Withdrew consent = 11
Transferred -out = 36
Retained in integrated care after at least 6 months = 430 (78.8%)
Multiple conditions
N=648
Did not meet eligibility criteria = 17
Declined with no reasons given = 4
Differed to discuss with family = 4
Approached late/no time = 8
Differed with no details given=3
Other reasons = 6
Enrolled into the study
N= 606
Did not receive integrated care =24
Received Integrated care for at least 6 months = 582
Loss to follow =24
Died=11
Withdrew consent = 1
Transferred out = 17
Retained in integrated care after at least 6 months = 529 (90.9%)

Figure 2.

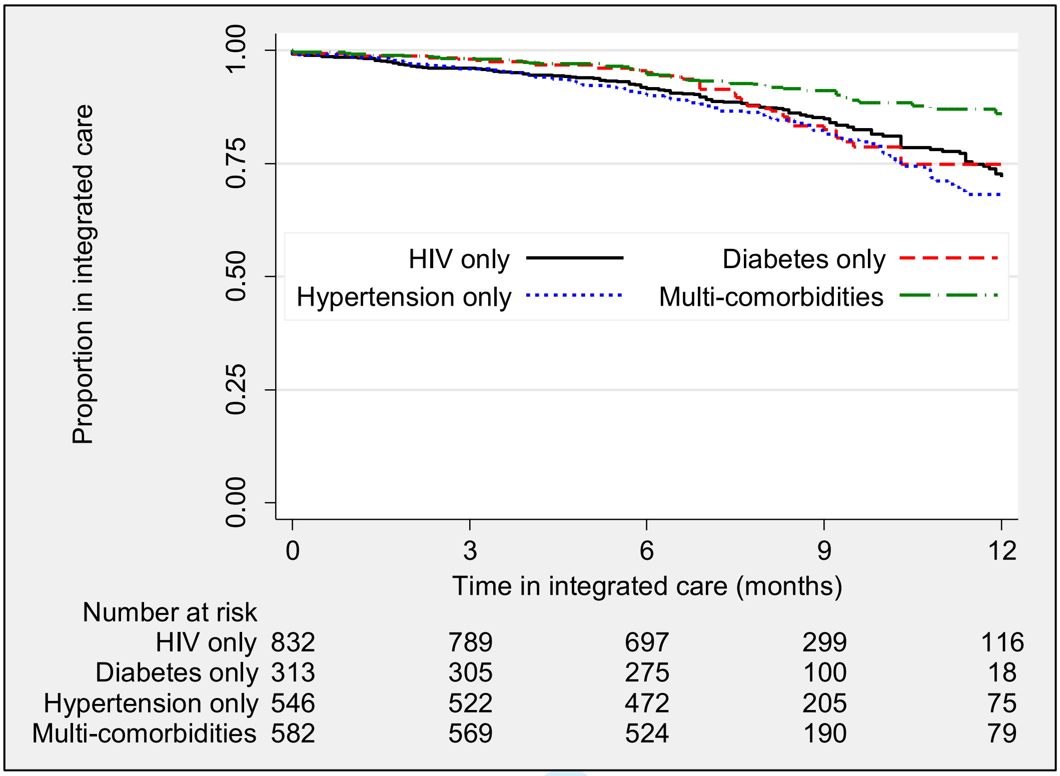
Proportion in integrated care
1.00
0.75
0.50
0.25
0.00
HIV only
Diabetes only
Hypertension only
Multi-comorbidities
0
3
6
9
12
Time in integrated care (months)
Number at risk
HIV only 832 789 697 299 116
Diabetes only 313 305 275 100 18
Hypertension only 546 522 472 205 75
Multi-comorbidities 582 569 524 190 79

Appendix

Description of HIV, diabetes and hypertension services provided at MOCCA study facilities.

Country	Facility name	Facility type	Notes *
Tanzania	Amana	Regional Hospital	Separate dedicated clinics run for HIV, diabetes and hypertension
	Shree Hindu Mandal	Hospital (Non Governmental Organisation)	Separate dedicated clinics for HIV, diabetes and hypertension; partial integration already occurring.
	Mwananyamala	Regional Hospital	Separate dedicated clinics run for HIV, diabetes and hypertension
	Mkuranga	District Hospital	Dedicated HIV clinic. Outpatient management of diabetes and hypertension
	Bunju	Dispensary	Dedicated HIV-clinic. No clinic for diabetes or hypertension
Uganda	Kiswa	Health Centre, level III	Dedicated HIV clinic. Separate outpatient screening for diabetes and hypertension patients who are then referred for management to a higher facility as necessary.
	Kisugu	Health Centre, level III	Separate HIV clinic. Separate outpatient screening for diabetes and hypertension patients who are then referred for management to a higher facility as necessary.
	TASO Uganda	Non Governmental Organisation	Providing management and care services to patients living with HIV. Includes management of diabetes and hypertension for patients with HIV-infection.
	Ndejje	Health Centre, level IV	Separate dedicated clinics for HIV, diabetes and hypertension
	Wakiso	Health Centre, level IV	Separate dedicated clinics for HIV, diabetes and hypertension.

* Separate dedicated clinics involved separate waiting and consultation areas, a separate pharmacy, and separate appointments and medical records systems. The patients were seen by dedicated physicians.

For peer review only
